# NF-κB across the Alzheimer disease spectrum: Context-dependent protective and pathogenic roles

**DOI:** 10.1016/j.ibneur.2026.08.008

**Published:** 2026-08-11

**Authors:** Ali Azargoonjahromi, Fatemeh Abutalebian, Hamide Nasiri

**Affiliations:** a Shiraz University of Medical Sciences, Shiraz, Iran; b Department of Biotechnology and Medicine, Islamic Azad University of Tehran Central Branch, Tehran, Iran; c Student Research Committee, School of Medicine, Zanjan University of Medical Science, Zanjan, Iran

**Keywords:** NF-κB, Alzheimer Disease, Neuroinflammation, Neuroprotection, Amyloid-β

## Abstract

Nuclear factor kappa B (NF-κB) has been implicated in both protective and detrimental processes in Alzheimer disease (AD), creating an apparent contradiction in the literature. Increasing evidence, however, indicates that these divergent effects reflect differences in cell type, molecular configuration, activation kinetics, disease state, and the surrounding pathological environment rather than a true biological paradox. Under transient and tightly regulated conditions, NF-κB can support neuronal survival, stress adaptation, antioxidant defense, and selected compensatory responses to amyloid-β pathology. In contrast, persistent or disease-conditioned signaling is more consistently associated with amyloidogenic processing, tau propagation, chronic neuroinflammation, impaired proteostasis, neuronal dysfunction, and neurovascular injury. The available evidence therefore supports an asymmetric framework in which protective effects are confined to relatively specific molecular and temporal settings, whereas sustained pathological signaling is supported across a broader range of disease-relevant models and cellular processes. This distinction argues against indiscriminate activation or global inhibition of NF-κB, either of which could disrupt physiological functions while failing to selectively suppress disease-driving pathways. Therapeutic strategies should instead target defined pathological NF-κB programs within the relevant cell type and disease context while preserving adaptive and homeostatic signaling. This review synthesizes the context-dependent roles of NF-κB in AD and provides a framework for reconciling its apparently opposing effects.

## Highlights

• NF-κB exerts context-dependent protective and pathogenic effects in Alzheimer’s disease (AD).
• Transient NF-κB signaling may support neuronal and glial adaptive responses.
• Persistent RelA/p65-biased signaling is linked to major AD-related pathologies.
• Human evidence reveals distinct patterns of impaired and exaggerated NF-κB signaling.
• Precision targeting may suppress pathological NF-κB while preserving homeostatic functions.

## 1 Introduction

Nuclear factor kappa B (NF-κB) is involved in several processes central to Alzheimer disease (AD), including neuronal stress responses, neuroinflammation, amyloid-β (Aβ) and tau pathology, synaptic dysfunction, and neurovascular injury (Kaltschmidt et al., 2024, Singh and Singh, 2020, Sun et al., 2022). Its role in AD, however, has been difficult to define because NF-κB signaling does not produce a single biological outcome. Under some conditions, it supports neuronal survival and adaptive stress responses, whereas under others it drives inflammatory and neurodegenerative processes. These apparently opposing effects are therefore better understood as context dependent rather than truly paradoxical; these findings suggest that the biological consequences of NF-κB depend on the signaling complex engaged, the responding cell, the initiating stimulus, and the duration and downstream consequences of activation.

This context dependence is a fundamental feature of the NF-κB signaling system. The NF-κB family comprises RelA/p65, RelB, c-Rel, p50/NFKB1, and p52/NFKB2, which can combine to form different homo- and heterodimers with distinct transcriptional properties. The activity of these dimers is further influenced by interacting cofactors, chromatin accessibility, post-translational modifications, and the timing and duration of signaling (Oeckinghaus and Ghosh, 2009). NF-κB activation occurs mainly through canonical and non-canonical pathways. Canonical signaling typically involves IκB kinase (IKK)-mediated release and activation of complexes containing RelA or c-Rel, whereas non-canonical signaling depends on NF-κB-inducing kinase (NIK), IKKα, processing of p100 to p52, and subsequent activation of RelB/p52 complexes (Sun, 2012). In AD, however, the non-canonical pathway remains relatively poorly characterized, as most mechanistic studies have focused on canonical NF-κB signaling (Kaltschmidt et al., 2024, Singh and Singh, 2020, Sun et al., 2022).

These distinctions are essential when interpreting experimental evidence, because commonly used readouts capture different stages of NF-κB signaling and should not be treated as equivalent. IκB degradation indicates release of NF-κB from cytoplasmic inhibition, whereas nuclear p65 accumulation reflects translocation, κB-DNA binding demonstrates interaction with regulatory DNA, and target-gene induction provides stronger evidence that transcriptional activity has actually occurred. Similar caution is required when interpreting IKKβ manipulation. Although IKKβ is a central component of canonical NF-κB signaling, it also phosphorylates substrates outside the NF-κB pathway and can independently influence autophagy, proteostasis, and cell-death processes (Antonia et al., 2021, Häcker and Karin, 2006). Therefore, findings based solely on IKKβ manipulation or nuclear localization of p65 should be interpreted cautiously, because neither observation by itself identifies the specific NF-κB transcriptional program responsible for the biological effect. IKKβ can regulate processes beyond canonical NF-κB transcription, and nuclear p65 does not reveal which NF-κB dimer is active or which target genes are functionally involved. Mechanistic evidence is therefore strongest when studies directly connect a defined NF-κB complex to a specific transcriptional target and biological outcome using complementary approaches such as gain- or loss-of-function experiments, promoter-binding or reporter assays, pathway inhibition, and rescue experiments.

Cellular context provides another important explanation for the apparently conflicting effects of NF-κB in AD. NF-κB signaling occurs in neurons, microglia, astrocytes, endothelial cells, and pericytes, but its biological consequences differ substantially across these cell types. In microglia, for example, NF-κB activation can promote the release and propagation of seeding-competent tau (Wang et al., 2022a). In astrocytes, its effects appear more context dependent: NF-κB may support selected adaptive responses once pathology is established, whereas persistent activation in a relatively healthy brain can disturb inflammatory and metabolic homeostasis (Jong Huat et al., 2024). NF-κB also contributes to neurovascular dysfunction. In APOE4 carriers, impaired suppression of the pericytic cyclophilin A (CypA)–NF-κB–MMP9 pathway can promote blood–brain barrier breakdown and cerebral hypoperfusion (Bell et al., 2012). These findings show why NF-κB activation cannot be classified as uniformly protective or pathogenic on the basis of a single molecular marker or outcome. Likewise, an improvement in one endpoint, such as reduced plaque burden or altered cytokine expression, does not necessarily indicate an overall beneficial effect. A meaningful interpretation therefore requires consideration of the molecular pathway involved, the responding cell type, the underlying pathological state, and the broader functional consequences.

Viewed within this framework, the dual effects of NF-κB in AD appear to be context dependent but not equally supported across biological settings. Protective effects are most consistently observed under relatively restricted conditions, particularly during transient neuronal stress responses, c-Rel-dependent survival signaling, and selected compensatory functions in glial cells. In contrast, sustained disease-associated IKK–RelA signaling has been linked across a wider range of experimental models to amyloidogenic processing, amplification of neuroinflammation, tau propagation, and neurovascular dysfunction.

Accordingly, this review evaluates primary studies in relation to their cellular and molecular context and the strength of the causal evidence they provide. Particular emphasis is placed on cell-specific gain- and loss-of-function studies and on experiments that use pathway inhibition, genetic manipulation, or rescue approaches to establish mechanistic links. The central question is therefore not whether NF-κB is inherently protective or pathogenic in AD, but rather which NF-κB signaling program is engaged, in which cell type, under what pathological conditions, and with what biological consequences.

## 2 Literature Identification and Appraisal of Evidence

The literature search was designed to identify mechanistically informative studies rather than every intervention in which NF-κB changed as a secondary outcome. PubMed/MEDLINE, Europe PMC, publisher databases, and backward and forward citation searches were examined iteratively through 3 August 2026. Search terms combined *Alzheimer’s disease*, *mild cognitive impairment*, *amyloid-β*, *amyloid precursor protein*, *amyloid* and *tau* models, and major NF-κB pathway components, including *NF-κB, RelA/p65, NFKB1/p50, NFKB2/p52, c-Rel, IκB kinase, IκB,* and NF-κB essential modulator (NEMO). Reviews, editorials, comments, patents, corrections, and retracted records were excluded from the primary synthesis.

The final evidence set comprised 71 primary studies selected for their contribution to human disease relevance, direct pathway manipulation, promoter-level or molecular mechanisms, pathway blockade or rescue, cell-specific causality, or clarification of protective versus detrimental effects. Selection was therefore purposive and mechanism centered. The 71 studies should be interpreted as an analytically defined evidence set rather than as a denominator for estimating the prevalence of protective or pathogenic NF-κB effects.

For each study, we extracted the experimental model and cell type, species or human source, age and sex when reported, Aβ or tau species and exposure conditions, pathway component and manipulation, sample size, principal methods, NF-κB readout, disease-relevant outcome, mechanistic direction, causal strength, and major limitations. Numerical details were included only when verifiable in the primary source; unavailable information was recorded as not reported. This was particularly important for Aβ studies, in which peptide species, aggregation state, concentration, exposure duration, and accompanying stimuli can substantially alter NF-κB responses.

Evidence was classified according to the degree of causal inference supported by the study design. Tier A included cell-relevant in-vivo genetic manipulations testing necessity or sufficiency for an organism-level phenotype. Tier B included direct genetic or pharmacological perturbations strengthened by promoter mutation, pathway blockade, epistasis, or rescue. Tier C comprised human association studies and mechanistic experiments demonstrating disease relevance without organism-level causality, whereas Tier D included localization studies and secondary pathway measurements without direct tests of necessity or sufficiency. These tiers reflect causal resolution rather than overall study quality.

The evidence base was dominated by experimental and amyloid-focused studies. Forty-six studies included an in-vitro component, 40 an in-vivo animal, fly, or nematode component, and 17 participants, postmortem tissue, or human-derived cells; categories overlapped. Aβ exposure or injection was used in 49 studies, compared with seven APP/PS1 studies, two 5xFAD studies, and relatively few APP knock-in or dedicated tau models. Neurons were examined in 42 studies, microglia in 15, astrocytes in 13, and endothelial or pericyte compartments in six. Pathway definition was also uneven: 49 studies reported generic NF-κB activity, 21 examined RelA/p65, ten p50, seven IKK/IKKβ, and only one centered on c-Rel. Thus, the literature is rich in acute Aβ- and RelA-associated findings but comparatively sparse for c-Rel, non-canonical signaling, multicellular interactions, vascular biology, aging, and sporadic AD.

Of the 71 studies, 41 were judged predominantly detrimental, ten beneficial or potentially homeostatic, nine mixed, two beneficial at the IKKβ level without demonstrated mediation by canonical NF-κB transcription, and nine directionally unresolved. Eight studies met Tier A criteria, 25 Tier B, 18 Tier C, and 20 Tier D. These counts describe the composition of the selected literature and should not be interpreted as pooled effect estimates or as evidence that pathogenic NF-κB states are biologically more prevalent. Direct genetic, promoter-level, blockade, or rescue experiments were therefore given greater interpretive weight than secondary changes in NF-κB markers after indirect interventions. (Table 1)

**Table 1:** Comparative architecture of the 71-study evidence base.

Dimension	Distribution	Critical implication
Publication period	1996–2005: 18 studies; 2006–2015: 26; 2016–2026: 27	Later studies increasingly incorporated cell-specific genetics, tau pathology, APOE4, and human-derived models.
Experimental design*	46 in vitro; 40 in vivo; 17 involving participants, postmortem tissue, or human-derived cells	Mechanistic evidence is extensive, but no human interventional study directly tested selective NF-κB modulation.
Disease model	49 used Aβ exposure or injection; 7 APP/PS1; 2 5xFAD; few APP knock-in or dedicated tau models	Acute amyloid stress dominates the literature.
Cell or tissue compartment*	42 neuronal; 15 microglial; 13 astrocytic; 6 endothelial or pericytic	Neuronal models predominate; multicellular and vascular interactions are underrepresented.
Causal tier	Tier A: 8; Tier B: 25; Tier C: 18; Tier D: 20	Causal weight varies substantially across studies.
Biological direction	41 detrimental; 10 beneficial or potentially homeostatic; 9 mixed; 2 beneficial at the IKKβ level with canonical NF-κB mediation unresolved; 9 unresolved	Directional counts reflect study composition, not biological prevalence.
Pathway definition*	49 generic NF-κB activity; 21 RelA/p65; 10 p50; 7 IKK/IKKβ; 1 centered on c-Rel	Broad conclusions are often based on RelA/p65 or nonspecific pathway measures.

* Categories overlap because individual studies could include more than one design, cell type, or pathway measure. All counts are descriptive and should not be interpreted as pooled effect estimates.

Several limitations should be acknowledged. This was a narrative rather than systematic review; the protocol was not prospectively registered, screening and data extraction were not independently duplicated, and formal domain-based risk-of-bias tools were not applied. Considerable heterogeneity in experimental models, exposure conditions, NF-κB measurements, and outcomes also precluded quantitative pooling. Publication bias, model selection, and preferential reporting of statistically significant or mechanistically favorable findings may therefore have influenced the available evidence.

Table 2 provides the study-level evidence map, including study design, population or model, exposure conditions, comparator, principal methods and outcomes, key findings, and major limitations. This structured map was used to distinguish direct causal evidence from association and to support the narrative synthesis of broader mechanistic patterns rather than presenting the 71 studies as isolated summaries. (Table 2)

**Table 2:** Study-level characteristics and principal findings of NF-κB-related studies across the AD spectrum.

Author(s) / Date	Population/model	Intervention/exposure	Comparator	Study design/setting	Main findings	Main limitation	Ref
(Sudo, Sung et al. 2026)	H4 cells; primary mouse glia predominantly astrocytes; seven-month-old male wild-type mice; 14–16-month-old female AppNL-G-F knock-in and AppNL-G-F; Klk7-null mice; Mayo and MSBB human AD transcriptomic cohorts.	H4 cells: IKK−16 2 µM or JSH−23 30 µM for 24 h with 20 nM Aβ40; primary glia: IKK−16 with 48-h Aβ-degradation assay. Mice: 4 µL of 100 µM IKK−16 at 0.3 µL/min into each hippocampus, with contralateral vehicle; collection at 24 h (Klk7) or 48 h (Aβ).	transgenic/modified vs wild-type; loss-of-function vs matched control; inhibitor/antagonist vs vehicle/untreated; treated hippocampus vs contralateral vehicle.	Human in silico/omics; Human in vitro; Rodent in vitro; Rodent in vivo	Both NF-κB inhibitors increased KLK7 expression and extracellular Aβ degradation without affecting cell viability or Aβ uptake. Local hippocampal IKK−16 reduced soluble and insoluble Aβ and decreased plaque size, but not plaque number. These Aβ-lowering effects disappeared in Klk7-null mice.	48-h local inhibition cannot establish chronic efficacy, cognition, cell specificity or preservation of other NF-κB functions; KLK7 is not astrocyte-exclusive.	(Sudo et al., 2026)
(Ye, Deng et al. 2026)	APP/PS1 mice across disease ages; Aβ-exposed primary mouse astrocytes, including Hdac7-floxed cultures; astrocyte-neuron conditioned-medium experiments.	Astrocyte-directed AAV-GfaABC1D HDAC7 gain or loss, Hdac7 excision, IKK blockade, and intraperitoneal TMP195. Vector titre, TMP195 dose, group size, sex, and schedule: NR.	HDAC7 gain-of-function, loss-of-function, and IKK blockade vs matched vector, floxed, or vehicle controls.	Rodent in vitro; Rodent in vivo	Aβ increased astrocytic HDAC7, which activated IKK/NF-κB. HDAC7 knockdown, IKK inhibition, or TMP195 reduced astrocyte-mediated neuronal injury and improved cognitive outcomes.	The APP/PS1 setting and incomplete dosing limit translation to sporadic AD.	(Ye et al., 2026)
(Zhang, Wang et al. 2026)	Aβ1–42-treated SH-SY5Y neuronal cells, APP/PS1 mice, and human-cell/clinical corroborative material reported by the authors.	TLR2 knockdown in cells and sh-TLR2 delivery in APP/PS1 mice; NF-κB overexpression and NLRP3 reactivation as reversal tests. Aβ concentration, vector dose, route, and treatment duration: NR.	TLR2 knockdown vs control; NF-κB overexpression or NLRP3 reactivation as reversal conditions.	Human in vitro; Rodent in vivo	TLR2 knockdown reduced inflammation, neuronal pyroptosis, brain pathology, and behavioral impairment. Re-activating NF-κB or NLRP3 partially removed this protection.	Neuronal-line and familial-transgenic models do not model the aged sporadic brain.	(Zhang et al., 2026a)
(Du, Yu et al. 2025)	PINK1-manipulated AD-model mice and Aβ-producing neuronal cell lines.	Neuronal PINK1 gain and loss plus ROS scavenging; doses, treatment duration, animal age, sex, and group size: NR.	PINK1 gain- and loss-of-function vs matched controls; reactive-oxygen-species scavenging as a pathway-interruption condition.	In vitro; Rodent in vivo	PINK1 loss increased mitochondrial reactive oxygen species, p50/p65 phosphorylation, secretase activity, and Aβ production. Scavenging reactive oxygen species prevented these downstream changes.	NF-κB necessity was not directly tested; pathway activation could remain a correlated intermediate.	(Du et al., 2025)
(Liu, He et al. 2025)	Public human AD transcriptomic datasets, Aβ1–42-treated SH-SY5Y cells, LPS-stimulated HMC3 microglia, and APP/PS1 mice.	SLAMF8 overexpression and NINJ2 knockout or suppression; Aβ/LPS exposure and in-vivo manipulation doses and durations: NR.	SLAMF8 overexpression with or without NINJ2 loss vs matched controls.	Human in silico/omics; Human in vitro; Rodent in vivo	SLAMF8 increased inflammatory and oxidative injury by activating TLR4/NF-κB through NINJ2. Removing NINJ2 prevented these effects in the tested cell and mouse models.	The mixed toxin, immortalized-cell, and familial-mouse systems complicate attribution to one disease stage.	(Liu et al., 2025)
(Vazquez-Coto, Perez-Oliveira et al. 2025)	Spanish case-control cohort of 639 late-onset AD cases and 500 cognitively healthy controls.	Genotyping of functional/common NFKB1, NFKBIA, and NFKBIZ variants; no experimental exposure.	AD vs cognitively healthy controls.	Human observational/genetic	The NFKB1 rs7667496 CC genotype and a related haplotype were associated with higher late-onset AD risk. The study did not test how the variant alters NF-κB function.	Population stratification, multiple testing, linkage, and lack of independent functional replication constrain causal interpretation.	(Vazquez-Coto et al., 2025a)
(Asanomi, Kimura et al. 2024)	HEK293 cells with homozygous CRISPR/Cas9 knock-in of SHARPIN G186R; reported knock-in efficiency was below 1%.	Endogenous risk-variant knock-in then TNF-α stimulation; TNF-α dose and duration were NR in the accessible article summary.	SHARPIN G186R knock-in cells vs reference/wild-type cells.	Human in vitro	Introducing the SHARPIN G186R risk variant reduced stimulus-induced NF-κB signaling and accelerated extracellular Aβ secretion.	A single rare clone in non-neural cells and very low editing efficiency raise clonal and generalizability concerns.	(Asanomi et al., 2024)
(Hamilton, Kinscherf et al. 2024)	Primary rat and mouse hippocampal astrocytes, Aβ25–35 exposure, APP/PS1 mouse tissue, human AD tissue, and human iPSC-derived astrocytes.	Wild-type or ligand-binding-deficient FABP7 overexpression; Aβ concentration and exposure duration: NR.	Wild-type FABP7 vs ligand-binding-deficient FABP7; AD-model vs control tissue.	Human in vitro; Rodent in vitro; Rodent in vivo	FABP7 was increased in plaque-associated astrocytes in mouse and human AD tissue. Only ligand-binding-competent FABP7 induced the broad NF-κB-linked inflammatory gene program.	Monoculture activation and acute Aβ25–35 do not establish chronic in-vivo neurotoxicity.	(Hamilton et al., 2024a)
(Jong Huat, Camats-Perna et al. 2024)	Human bulk and cell-resolved AD transcriptomic data and postmortem brain; healthy mice with chronic astrocytic NF-κB activation; an AD mouse model with astrocytic pathway inhibition.	Chronic astrocyte-specific gain of NF-κB in healthy brain and inhibition in diseased brain; construct, dose, age, and duration details: NR.	Healthy vs AD human tissue/data; astrocytic NF-κB activation in healthy mice and inhibition in an AD model vs matched controls.	Human ex vivo/biospecimen; Human in silico/omics; Rodent in vivo	Sustained astrocytic NF-κB activation disrupted protein homeostasis and increased inflammation in healthy mice. In contrast, inhibiting astrocytic NF-κB in an AD model accelerated Aβ and tau accumulation.	Different backgrounds and intervention directions prevent a simple within-model stage comparison.	(Jong Huat et al., 2024)
(Wahl, Risen et al. 2024)	Nineteen-month-old wild-type mice as an aging model and two-month-old rTg4510 tau-transgenic mice.	A brain-penetrant nanoligomer cocktail directed at NF-κB and NLRP3 for four weeks; dose, route, sex balance, and group size: NR.	Nanoligomer-treated vs control mice across aged wild-type and rTg4510 models.	Rodent in vivo	A four-week nanoligomer treatment targeting both NF-κB and NLRP3 reduced neuroinflammation and improved cognitive and tau-related outcomes in aging and tauopathy models.	The cocktail targets two nodes simultaneously and does not isolate NF-κB-specific mediation.	(Wahl et al., 2024)
(Zhao, Liu et al. 2024)	Plasma from patients with AD, APP/PS1 and 5xFAD mice, and EOC20 microglia in homeostatic or LPS/IFN-γ-activated states.	miR−25802 overexpression or inhibition with KLF4 restoration as a rescue; oligonucleotide, LPS/IFN-γ, and in-vivo doses and durations: NR.	miR−25802 gain/loss vs controls; KLF4 restoration as rescue; AD vs control plasma.	Human observational; Rodent in vitro; Rodent in vivo	miR−25802 promoted an inflammatory microglial state, worsened amyloid pathology, and impaired cognition. Restoring KLF4 weakened these effects.	EOC20 cells and two familial amyloid models may overrepresent an activated microglial phenotype.	(Zhao et al., 2024a)
(Schnöder, Quan et al. 2023)	Neuron-specific IKKβ-deficient mice crossed with APP- and tau-transgenic lines, plus SH-SY5Y experiments.	Conditional neuronal Ikbkb deletion and cellular IKKβ manipulation; ages, sex, group sizes, and cell-treatment doses: NR.	Neuron-specific Ikbkb deletion vs floxed/control mice in APP and tau backgrounds.	Human in vitro; Rodent in vivo	Deleting neuronal IKKβ reduced BACE1, Aβ, phosphorylated tau, apoptosis, and inflammation. Cognitive improvement occurred in some, but not all, transgenic models.	Cognition diverged between APP and tau models, arguing against a single downstream mechanism.	(Schnöder et al., 2023)
(Arnaud, Benech et al. 2022)	Isogenic human iPSC lines carrying APOE3/3, APOE4/4, or APOE knockout differentiated into astrocytes, with AD-brain validation.	CRISPR-defined APOE genotypes, pharmacological NF-κB modulation, and TAGLN3 re-expression or supplementation; compound concentrations and durations: NR.	loss-of-function vs matched control; rescue/restoration vs intervention alone; isogenic APOE3/3 vs APOE4/4 vs APOE-null astrocytes.	Human in vitro	APOE4 reduced TAGLN3 and made human iPSC-derived astrocytes persistently more responsive to inflammatory NF-κB signaling. Restoring TAGLN3 or pharmacologically modifying the pathway reversed this state.	iPSC reprogramming attenuates aging and astrocyte monoculture omits plaque, microglial, and vascular feedback.	(Arnaud et al., 2022a)
(Asanomi, Shigemizu et al. 2022)	Whole-genome sequencing in 180 late-onset AD and 184 MCI participants, then association testing in 5043 cases and 11,984 controls.	Identification and functional testing of SHARPIN R274W; No therapeutic exposure.	AD/MCI vs controls; risk variant vs reference/wild-type condition.	Human observational/genetic	The SHARPIN R274W risk variant altered protein localization, weakened inducible NF-κB signaling, and was associated with increased late-onset AD risk.	Modest effect size and uncertain responsible cell type prevent therapeutic extrapolation.	(Asanomi et al., 2022a)
(Wang, Gu et al. 2022)	Aβ-treated SH-SY5Y neuronal cells and APP/PS1 mice.	IKKβ silencing or constitutive activation with autophagy and RIPK1-necroptosis manipulation; doses, vector titres, animal ages, and durations: NR.	IKKβ silencing vs constitutive activation and matched controls.	Human in vitro; Rodent in vivo	IKKβ activation improved autophagic flux, reduced RIPK1-mediated necroptosis and Aβ accumulation, and improved neuronal, pathological, and behavioral outcomes.	Canonical NF-κB transcription was not shown to mediate autophagy/necroptosis effects.	(Wang et al., 2022b)
(Wang, Gu et al. 2022)	APP/PS1 mice with hippocampal CA1 manipulation and Aβ1–42-treated N2a neuronal cells.	miR−155–5p inhibition, IKKβ knockdown, SKP2 manipulation, and adenoviral delivery; vector titre, Aβ dose, treatment duration, animal age, and sex: NR.	miR−155 inhibition, IKKβ knockdown, and SKP2 manipulation vs matched controls.	Rodent in vitro; Rodent in vivo	miR−155–5p stabilized IKKβ by suppressing SKP2. Inhibiting miR−155–5p or reducing IKKβ lowered Aβ deposition, tissue injury, and cognitive impairment.	Direct canonical NF-κB transcriptional mediation and vector-dose dependence were not resolved.	(Wang et al., 2022c)
(Wang, Fan et al. 2022)	Primary mouse microglia; young (three-month-old) and aged (8–11-month-old) PS19 tauopathy mice with Cx3cr1-CreERT2-mediated microglial Ikbkb deletion or constitutive activation; AD- and PSP-derived tau preparations and tau-biosensor cells.	Primary microglia: full-length tau fibrils 2 µg/mL or K18/PL fibrils 2.5 µg/mL for 24 h; AD-tau 1 µg/mL after 2-h pretreatment with TPCA−1 0.1 µM. In vivo: unilateral hippocampal K18/PL, 2 µL at 2.5 µg/µL for loss-of-function or 2 µL at 0.2 µg/µL for gain-of-function, assessed after one month; chronic PS19 outcomes at 8–10 months.	Microglial Ikbkb deletion vs constitutive activation and matched controls; tau-exposure and TPCA−1 conditions.	Rodent in vitro; Rodent in vivo	Microglial NF-κB activation increased the release of tau capable of seeding new aggregates and promoted tau spread and cognitive decline. Microglial IKKβ deletion reduced propagation, improved autophagy and cognition, and normalized microglial gene expression, even though neuronal tau inclusions increased.	PS19 overexpresses mutant P301S tau; long-term consequences of retained inclusions and exact causal export machinery remain unresolved.	(Wang et al., 2022a)
(Chen, Zhang et al. 2021)	Mice receiving intracerebroventricular Aβ1–42 and Aβ-treated HT22 hippocampal cells.	Hippocampal DJ−1 shRNA, 8 × 10^4 viral particles per mouse for seven days, plus DJ−1/IKKβ/pVHL plasmid or shRNA manipulations in HT22 cells.	DJ−1, IKKβ, and pVHL gain/loss conditions vs matched plasmid or shRNA controls.	Rodent in vitro; Rodent in vivo	The DJ−1/pVHL pathway supported IKKβ-dependent autophagy and reduced phosphorylated tau, neuronal injury, and behavioral impairment.	Acute Aβ injection is not progressive AD and the benefit was not proven to require nuclear NF-κB transcription.	(Chen et al., 2021a)
(Lei, Liu et al. 2021)	Serum from 112 patients with AD and 101 healthy controls; commercial human hippocampal neurons exposed to Aβ25–35.	NF-κB knockdown, TIGAR overexpression, and miR−146a−5p manipulation/rescue; Aβ concentration and exposure duration: NR.	AD vs control serum; loss-of-function vs matched control; gain-of-function vs vector/baseline; rescue/restoration vs intervention alone.	Human observational/genetic; Human ex vivo/biospecimen; Human in vitro	NF-κB increased miR−146a−5p, which suppressed TIGAR and increased oxidative stress and neuronal pyroptosis. Restoring TIGAR or reducing the pathway attenuated these effects.	Serum and acute peptide-treated neurons cannot establish the brain source or temporal order in AD.	(Lei et al., 2021)
(Yang, Magnutzki et al. 2021)	APP23 mice with conditional astrocytic IKK2/NF-κB activation, with primary astrocyte and microglial phenotyping.	Astrocyte-specific IKK2 activation; induction timing, animal age, sex, group size, and activation magnitude: NR.	Astrocyte-specific IKK2/NF-κB activation vs nonactivated controls; direct microglial activation as comparator.	Rodent in vitro; Rodent in vivo	Astrocyte-specific IKK2/NF-κB activation increased gliosis but reduced the number and size of compact plaques and shifted nearby microglia toward an Aβ-handling state.	Soluble Aβ species and neuronal function are needed before plaque reduction is called net protection.	(Yang et al., 2021a)
(Asanomi, Shigemizu et al. 2019)	Whole-exome sequencing of 202 late-onset AD cases lacking APOE ε4, followed by 4563 cases and 16,459 controls plus cellular functional assays.	Functional characterization of rare SHARPIN G186R; No therapeutic exposure.	AD/MCI vs controls; risk variant vs reference/wild-type condition.	Human observational/genetic	The rare SHARPIN G186R variant weakened stimulated NF-κB responses and was associated with substantially higher late-onset AD risk.	Rarity, ancestry, and uncertain brain-cell locus limit generalization.	(Asanomi et al., 2019a)
(Yamaguchi, Ayaki et al. 2019)	postmortem hippocampus from 21 autopsies spanning AD, ALS with optineurin pathology, other neurodegenerative diseases, and controls.	No exposure; comparative neuropathological staining.	AD/disease vs control tissue.	Human ex vivo/biospecimen	Phosphorylated p65 accumulated in granulovacuolar degeneration and tau-positive neurites, particularly in AD tissue.	Cannot distinguish active transcription from sequestration or determine whether phospho-p65 precedes tau pathology.	(Yamaguchi et al., 2019a)
(Li, Sibon et al. 2018)	Drosophila proteotoxicity screen initiated in a neuronal SCA3 eye model and tested in flies expressing human neuronal Aβ.	Astrocyte-specific inhibition of the Drosophila NF-κB homolog Relish; genetic driver strength and induction timing: NR.	Astrocyte-like glial Relish inhibition vs genetic-driver controls.	Invertebrate in vivo	Inhibiting Relish/NF-κB specifically in astrocyte-like glia delayed neurodegeneration and extended survival without directly changing neuronal Aβ expression.	Fly innate immunity and astrocyte biology only partially model mammalian AD.	(Li et al., 2018)
(Tanaka, Sabharwal et al. 2018)	Psen1-deficient mouse endothelial cells and cells with PS1 re-expression or overexpression.	PS1 gain/loss, γ-secretase inhibition, and manipulation of BCR/CK2α/p65 signaling; concentrations and durations: NR.	Psen1 loss, restoration, or overexpression with γ-secretase/pathway inhibition vs matched controls.	Rodent in vitro	Presenilin 1 assembled a BCR-CK2α-p65 complex and promoted p65 Ser529 phosphorylation, NF-κB reporter activity, and interleukin−6 expression independently of γ-secretase.	The study did not establish an AD phenotype or in-vivo vascular consequence.	(Tanaka et al., 2018)
(Shi, Hong et al. 2017)	APP/BACE1 promoter cell systems and an AD mouse model receiving hippocampal lentiviral BAG-1M.	BAG-1M overexpression and promoter-complex manipulation; lentiviral titre, animal age, dose, and follow-up: NR.	BAG-1M overexpression/promoter manipulation vs vector or promoter controls.	In vitro; Rodent in vivo	BAG-1M increased NF-κB activity at the BACE1 promoter, raising BACE1 expression, Aβ production, plaque burden, and memory impairment.	Supraphysiological BAG-1M and incomplete dosing information limit effect-size interpretation.	(Shi et al., 2017)
(Liao, Qi et al. 2016)	postmortem hippocampal, temporal, and frontal cortex from AD and control brains; Aβ1–42-treated SH-SY5Y and U87MG cells.	Aβ1–42 exposure and α3 nicotinic-acetylcholine-receptor siRNA; dose, duration, and human sample size: NR.	AD vs control postmortem tissue; α3-nAChR knockdown vs control siRNA in Aβ-exposed cells.	Human ex vivo/biospecimen; Human in vitro	p65 and inflammatory chemokines were increased in AD brain tissue and Aβ-treated neural cells. Reducing α3 nicotinic acetylcholine receptor expression further increased this inflammatory response.	Bulk tissue composition, acute Aβ exposure, and absent causal in-vivo testing restrict inference.	(Liao et al., 2016)
(Yang, Liu et al. 2016)	Primary mouse microglia basal or LPS.	Lrp1 knockdown or receptor-associated protein blockade, with JNK and NF-κB inhibitors; inhibitor and LPS concentrations and durations: NR.	loss-of-function vs matched control; inhibitor/antagonist vs vehicle/untreated.	Rodent in vitro	Loss or blockade of LRP1 increased JNK/NF-κB activity and made primary microglia more responsive to inflammatory stimulation.	The experiment did not include amyloid or an AD animal phenotype.	(Yang et al., 2016)
(Lian, Yang et al. 2015)	Aβ-exposed astrocyte-neuron systems, APP-transgenic mice, and human AD brain.	Astroglial NF-κB manipulation and neuronal C3a-receptor antagonism; Aβ, genetic, and antagonist doses/schedules: NR.	Astroglial NF-κB manipulation and neuronal C3aR antagonism vs matched controls.	Human ex vivo/biospecimen; In vitro; Rodent in vivo	Aβ-activated astrocytic NF-κB increased complement C3 release. C3 signaling through neuronal C3a receptors damaged dendrites, disrupted network function, and impaired cognition; receptor blockade reduced these effects.	Human evidence is cross-sectional and intervention details require full-text verification.	(Lian et al., 2015a)
(Quan, Yue et al. 2015)	APP Swedish-mutant SH-SY5Y cells and CL2006 Caenorhabditis elegans expressing human Aβ.	PRMT5 knockdown, E2F1 codepletion, and NF-κB or GSK3 inhibition; inhibitor concentrations and worm exposure schedules: NR.	PRMT5 knockdown with E2F1 codepletion or NF-κB/GSK3 inhibition vs matched controls.	Human in vitro; Invertebrate in vivo	PRMT5 loss activated an E2F1-NF-κB-GSK3β stress pathway and increased apoptosis. NF-κB or GSK3 inhibition reduced cell injury and age-related paralysis in the nematode model.	Cultured tumor cells and nematodes lack the mammalian glial and vascular context.	(Quan et al., 2015)
(Rangasamy, Corbett et al. 2015)	Five-month-old 5xFAD mice, with wild-type littermate controls.	Wild-type NEMO-binding-domain peptide or inactive mutant peptide, 0.1 mg/kg intranasally every other day for 30 days; a single-dose distribution experiment assessed brain entry at 30 min.	transgenic/modified vs wild-type; active peptide vs inactive mutant peptide.	Rodent in vivo	Intranasal NEMO-binding-domain peptide entered the brain, reduced canonical NF-κB signaling and gliosis, improved amyloid- and tau-related measures, and prevented memory impairment.	Small groups and broad NEMO/IKK inhibition leave cell specificity and preserved homeostatic signaling unresolved.	(Rangasamy et al., 2015a)
(Liu, Liu et al. 2014)	Six-month-old TgCRND8 amyloid mice with LysM-Cre-mediated conditional myeloid Ikbkb deletion; isolated microglia, bone-marrow-derived macrophages plus recruitment/internalization assays.	Genetic myeloid IKKβ deficiency. In vitro, oligomeric Aβ42 was tested at 1 or 10 µM in macrophage internalization assays; no in-vivo drug dose.	Myeloid Ikbkb deletion vs littermate controls; IKKβ-deficient vs control macrophages in Aβ internalization assays.	Rodent in vitro; Rodent in vivo	Myeloid IKKβ deletion reduced TNF and interleukin−1β, lowered selected Aβ measures, preserved synaptic proteins, and improved cognition. Microglial recruitment and Aβ internalization were preserved or increased.	LysM targeting includes peripheral myeloid cells, the responsible NF-κB dimer was not isolated, and greater uptake did not by itself prove complete lysosomal degradation.	(Liu et al., 2014)
(Ohta, Tremblay et al. 2014)	Temporal cortex from 12 MCI, 12 AD, and 12 age-matched control cases; four MCI cases with episodic-memory deficits were analyzed in depth.	No treatment; postmortem immunoprecipitation, immunoblotting, and immunofluorescence.	MCI and AD tissue vs age-matched controls.	Human ex vivo/biospecimen	A nuclear TDP−43-p65 complex and phosphorylated p65 were detected in a small subset of MCI cases with episodic-memory impairment.	The highlighted signal in four MCI cases is exploratory and cross-sectional.	(Ohta et al., 2014)
(Park, Kook et al. 2014)	bEnd.3 mouse brain endothelial cells, astrocyte-endothelial contact/polarity models, and 5xFAD mouse brain.	Aβ1–42 exposure, a RAGE-neutralizing antibody, and NF-κB inhibition; concentrations, duration, animal age, and group size: NR.	Aβ-exposed vs untreated endothelial models; RAGE neutralization or NF-κB inhibition vs control.	Rodent in vitro; Rodent in vivo	Aβ1–42 activated endothelial RAGE-NF-κB signaling and reduced P-glycoprotein. Blocking RAGE or NF-κB prevented transporter loss.	An immortalized endothelial line and familial amyloid model may not capture aged human BBB physiology.	(Park et al., 2014)
(Rolova, Puli et al. 2014)	Nfkb1/p50-knockout primary microglia, intrahippocampal LPS models, and p50-deficient mice crossed with APdE9 amyloid mice.	Constitutive p50 loss with LPS or Aβ challenges; challenge doses, ages, sex, and group sizes: NR.	Nfkb1/p50-null vs wild-type microglia and mice under acute and chronic inflammatory/Aβ conditions.	Rodent in vitro; Rodent in vivo	Loss of p50 increased microglial Aβ uptake and modestly reduced amyloid burden, but it also intensified chronic inflammatory gene expression and leukocyte infiltration.	Genetic deletion reveals a true trade-off—more uptake/slightly less Aβ but worse inflammation—although lifelong p50 loss permits developmental and systemic compensation.	(Rolova et al., 2014)
(Shi, Zhu et al. 2014)	HT22 mouse hippocampal cells and isolated mitochondria exposed to Aβ.	Aβ treatment and subcellular pathway perturbation; peptide species, concentration, exposure duration, and inhibitor doses: NR.	Aβ-exposed vs untreated cells/mitochondria with pathway perturbation.	Rodent in vitro; Subcellular/cell-free	Aβ activated an IκB/NF-κB system within mitochondria and was associated with impaired respiratory-chain function and mitochondrial dysfunction.	Immortalized cells and isolated mitochondria do not establish organism-level neurodegeneration.	(Shi et al., 2014a)
(Ascolani, Balestrieri et al. 2012)	Mitogen-stimulated peripheral-blood mononuclear cells from 12 patients with AD and 12 age-matched controls.	Ex-vivo mitogenic stimulation; mitogen identity/concentration and incubation duration: NR.	AD vs age-matched control PBMCs.	Human ex vivo	Mitogen-stimulated peripheral blood mononuclear cells from patients with AD showed greater NFKB1 expression, p50/p65 DNA binding, and target-gene expression. Several measures correlated with cognitive severity.	The small blood cohort is vulnerable to medication and systemic comorbidity and is not a direct brain readout.	(Ascolani et al., 2012)
(Bell, Winkler et al. 2012)	Human APOE2-, APOE3-, or APOE4-targeted-replacement mice and Apoe-null mice, with pericyte/vascular analyses.	Genetic CypA ablation and pharmacological CypA-pathway inhibition; compound dose and treatment schedule: NR.	APOE2, APOE3, APOE4, and Apoe-null mouse groups; loss-of-function vs matched control; inhibitor/antagonist vs vehicle/untreated.	Rodent in vivo (human APOE targeted-replacement)	APOE4 failed to suppress the pericyte CypA-NF-κB-MMP9 pathway. This caused blood-brain barrier leakage, reduced cerebral blood flow, and preceded neuronal and synaptic dysfunction.	Mice model APOE-dependent barrier injury rather than the complete Alzheimer spectrum.	(Bell et al., 2012)
(Chami, Buggia-Prévot et al. 2012)	HEK293 cells with or without Swedish-mutant APP expression.	Physiological vs supraphysiological Aβ exposure plus canonical and alternative NF-κB kinase modulators; exact concentration ranges and durations: NR.	Physiological vs supraphysiological Aβ conditions in cells with or without Swedish-mutant APP.	Human in vitro	At lower Aβ levels, NF-κB reduced APP and secretase expression. At higher Aβ levels, the direction reversed and NF-κB increased the same amyloid-producing machinery.	Non-neural HEK293 cells and incomplete exposure levels limit physiological extrapolation.	(Chami et al., 2012a)
(Chen, Zhou et al. 2012)	AD and control brain material plus neuronal/promoter systems, including RelA-deficient cells.	p65 expression, TNF stimulation, NSAID/NF-κB modulation, BACE1-promoter κB-site mutation, and RelA loss; doses and durations: NR.	AD/disease vs control tissue; loss-of-function vs matched control; wild-type vs mutated/deleted κB promoter.	Human ex vivo/biospecimen; In vitro	RelA/p65 and BACE1 were increased in AD brain tissue. Mutating the BACE1 κB site or removing RelA prevented promoter activation and reduced BACE1 expression, APP processing, and Aβ production.	Pharmacological effects and bulk human expression do not prove in-vivo neuronal necessity.	(Chen et al., 2012a)
(Guglielmotto, Monteleone et al. 2012)	SH-SY5Y and NT2 neuronal cells exposed to Aβ1–42, with postmortem AD-brain corroboration.	Aβ1–42 and NF-κB inhibition; peptide and inhibitor concentrations and treatment duration: NR.	Aβ1–42-exposed vs control neuronal cells; NF-κB inhibition vs vehicle; AD vs control tissue.	Human ex vivo/biospecimen; Human in vitro	Aβ1–42 activated NF-κB, reduced UCH-L1, and impaired cathepsin D-dependent lysosomal degradation of BACE1, allowing BACE1 to persist longer.	Rests largely on acute tumor-cell models; human tissue cannot establish temporal ordering.	(Guglielmotto et al., 2012a)
(Gonzalez-Velasquez, Reed et al. 2011)	Cultured cerebral microvascular endothelial monolayers exposed to Aβ1–40 in soluble, monomeric, or fibrillar states.	Aggregate-state and concentration comparisons extending to physiologically relevant low concentrations, with NF-κB blockade; exact range and inhibitor dose: NR.	inhibitor/antagonist vs vehicle/untreated; Aβ aggregation-state comparisons; dose/time comparisons.	In vitro	Soluble Aβ aggregates, more than monomeric or fibrillar forms, activated endothelial NF-κB and increased barrier permeability, leukocyte adhesion, and transmigration.	Monoculture lacks pericyte, astrocyte, flow, and chronic-aging influences.	(Gonzalez-Velasquez et al., 2011)
(Kauppinen, Suh et al. 2011)	hAPP-J20 mice crossed with Parp1-null mice, assessed at six months; intracerebral Aβ1–42 injection and primary microglia/neuronal cocultures.	Germline PARP1 loss and Aβ challenge; injected peptide and culture concentrations: NR.	Parp1-null vs wild-type hAPP-J20 mice and microglia; Aβ-challenged vs control cultures.	Rodent in vitro; Rodent in vivo	PARP1 deficiency reduced Aβ-induced microglial activation, neuronal injury, and cognitive impairment without reducing microglial Aβ uptake.	Germline deletion affects multiple cells and developmental processes.	(Kauppinen et al., 2011a)
(Blanco, Álvarez et al. 2010)	Primary human astrocytes and human astrocytoma cell lines exposed to Aβ42; conditioned medium was applied to neurons.	Aβ42, IκB overexpression, p65 overexpression, PDTC, and COX−2-promoter deletion constructs; exact doses and durations: NR.	Aβ-exposed astrocytes with IκB/p65/promoter manipulations vs matched controls; conditioned medium vs control medium.	Human in vitro	Aβ activated NF-κB-dependent COX−2 expression and prostaglandin E2 release in human astrocytes. Conditioned medium from these astrocytes was toxic to neurons.	Acute Aβ and tumor lines may exaggerate reactive signaling.	(Blanco et al., 2010a)
(Cui, Li et al. 2010)	Sixty-six control and AD brain samples plus primary human astroglia subjected to IL-1β, Aβ42, or oxidative stress.	Stress exposure, miR−146a promoter constructs, curcumin, PDTC, CAY10512, and anti-miR−146a; concentrations and durations: NR.	AD vs control brain; stressed vs unstressed astroglia; anti-miR/inhibitor conditions vs controls.	Human ex vivo/biospecimen; Human in vitro	NF-κB-induced miR−146a reduced IRAK1 and increased IRAK2 in stressed astroglia and AD brain tissue, helping maintain inflammatory signaling after the initial stress.	Mixed postmortem tissue and multiple pharmacological agents complicate cell-specific causality.	(Cui et al., 2010)
(Zhang, Luhrs et al. 2009)	APPswe/PS1dE9 mice and wild-type littermates, treated from seven to twelve months of age.	Pyrrolidine dithiocarbamate (PDTC), 50 mg/kg intraperitoneally once daily for five months.	transgenic/modified vs wild-type; inhibitor/antagonist vs vehicle/untreated.	Rodent in vivo	Long-term PDTC treatment reduced astrogliosis and inflammatory mediators but increased cerebral Aβ42, indicating a trade-off between inflammation and amyloid handling.	More Aβ42—yet PDTC is nonspecific and systemic treatment obscures the responsible cell.	(Zhang et al., 2009)
(Kawamoto, Lepsch et al. 2008)	Primary rat cerebellar mixed-cell cultures.	Aβ1–40 at 1 or 2 µM for 6, 12, or 24 h; receptor/pathway inhibitors were added 20 min before Aβ. The clearest activation occurred with 1 µM for 12 h.	Aβ dose/time conditions with receptor/pathway inhibitors vs untreated or vehicle controls.	Rodent in vitro	Aβ activated both p50/p65 and p50/p50 dimers through an NMDA-receptor-sensitive pathway. The study mapped the signaling route but did not determine whether it protected or injured cells.	Mixed cerebellar neonatal cultures are remote from aged hippocampal AD and outcome direction was not established.	(Kawamoto et al., 2008a)
(Lukiw, Zhao et al. 2008)	postmortem AD and control brain plus primary human neural cells stressed with IL-1β, Aβ42, or oxidative injury.	miR−146a promoter manipulation, PDTC, CAY10512, and anti-miR−146a; stressor and inhibitor doses and durations: NR.	AD vs control brain; stressed vs unstressed cells; promoter, inhibitor, and anti-miR rescue conditions.	Human ex vivo/biospecimen; Human in vitro	NF-κB-sensitive miR−146a was increased in AD brain and stressed human neural cells and reduced complement factor H, an inhibitor of complement-mediated inflammation.	Postmortem association and acute stressed cultures do not define its net role over disease time.	(Lukiw et al., 2008)
(Tan, Schedl et al. 2008)	Drosophila expressing human Aβ42 in the eye/neuronal tissue.	Genetic loss- and gain-of-function across Toll, NF-κB, and downstream innate-immune components; driver strength and developmental timing: NR.	loss-of-function vs matched control; gain-of-function vs vector/baseline.	Invertebrate in vivo	Reducing Toll-NF-κB signaling suppressed Aβ42-induced neurodegeneration in flies, whereas increasing the pathway worsened the phenotype.	The fly Toll/Relish system and developmental eye phenotype cannot model mammalian adaptive immunity, microglia, or cognition.	(Tan et al., 2008a)
(Ait-Ghezala, Volmar et al. 2007)	HEK293 cells coexpressing Swedish-mutant APP and CD40.	CD40 ligand, NF-κB inhibitors, and pathway-targeting siRNAs; concentrations and incubation times: NR.	CD40 ligand with NF-κB inhibitors or siRNAs vs untreated/control-transfected cells.	Human in vitro	CD40 ligand increased APP metabolites and Aβ production. NF-κB inhibitors and pathway-targeting siRNAs reduced this response.	Receptor and APP overexpression in non-neural cells may create supraphysiological signaling.	(Ait-Ghezala et al., 2007a)
(Bourne, Ferrari et al. 2007)	Neuronal cultures and resting or activated astrocytes carrying BACE1-promoter reporters.	Aβ exposure, cell activation, and mutation of the BACE1 κB element; peptide concentration, activation stimulus, and timing: NR.	Resting vs Aβ-exposed neurons and resting vs activated astrocytes; wild-type vs mutated κB promoter.	In vitro	The same BACE1 κB element suppressed transcription in resting neurons but activated transcription in Aβ-exposed neurons and activated astrocytes.	Reporter systems do not model chromatin topology or whole-brain APP metabolism.	(Bourne et al., 2007a)
(Paris, Patel et al. 2007)	CHO cells overexpressing APP.	A panel of NF-κB inhibitors; compound identities, concentrations, and exposure durations were incompletely reported.	inhibitor/antagonist vs vehicle/untreated.	Hamster-derived in vitro	Several NF-κB inhibitors reduced Aβ40 and Aβ42 production in APP-overexpressing cells, although the specific pathway responsible was not established.	Non-neural cells and inhibitor off-target effects prevent precise causal assignment.	(Paris et al., 2007a)
(Schapansky, Olson et al. 2007)	Neurons expressing normal or AD-linked mutant presenilin−1.	Aβ and IP3-linked endoplasmic-reticulum Ca2 + release with NF-κB inhibition or activation; concentrations and durations: NR.	Wild-type vs AD-linked presenilin−1 neurons; NF-κB activation/inhibition conditions.	In vitro	Endoplasmic-reticulum calcium release activated NF-κB, reduced the pro-apoptotic protein CHOP, and protected neurons from Aβ toxicity, especially in cells expressing mutant presenilin 1.	The familial-PS1 acute-culture context may not persist in chronic sporadic disease.	(Schapansky et al., 2007a)
(Choi, Kim et al. 2006)	M3-muscarinic-receptor-expressing SH-SY5Y neuronal cells.	Oxotremorine-M with SN50, BAY11–7085, MG132, PDTC, quinazoline, TMB8, gadolinium, or SKF96365; exact concentrations and durations: NR.	Oxotremorine-M-treated vs untreated cells with calcium-entry/NF-κB inhibitors.	Human in vitro	Muscarinic receptor-driven calcium entry activated NF-κB and increased soluble APPα release, favoring non-amyloidogenic APP processing.	The large pharmacological panel has overlapping off-target effects and lacks an in-vivo amyloid endpoint.	(Choi et al., 2006a)
(Chen, Zhou et al. 2005)	Primary microglia-neuron cultures exposed to Aβ.	Nondegradable IκBα super-repressor, SIRT1 overexpression, and resveratrol; Aβ and resveratrol doses and treatment durations: NR.	Aβ-exposed microglia-neuron cocultures with IκBα super-repressor, SIRT1 overexpression, or resveratrol vs controls.	Rodent in vitro	Blocking microglial NF-κB with a nondegradable IκBα construct reduced Aβ-induced neuronal death. SIRT1 overexpression or resveratrol produced similar protection by reducing p65 acetylation.	Acute coculture lacks chronic plaque and aging states.	(Chen et al., 2005a)
(Chong, Li et al. 2005)	Primary hippocampal neurons exposed to Aβ.	Erythropoietin across concentration conditions, with p65 RNA interference and tests of nuclear translocation; exact concentrations and timings: NR.	Erythropoietin-treated vs untreated Aβ-exposed neurons; p65 RNA interference or blocked translocation vs control.	Rodent in vitro	Erythropoietin protected primary hippocampal neurons from early and late Aβ-induced apoptosis. p65 knockdown or prevention of nuclear translocation abolished the protection.	A short-term pure-neuron system cannot show whether chronic glial activation offsets that benefit.	(Chong et al., 2005)
(Huang, Liu et al. 2005)	postmortem AD and control brain tissue.	No exposure; lectin-based enrichment, mass spectrometry, and immunoblot validation.	AD/disease vs control tissue.	Human ex vivo/biospecimen	The NF-κB precursor p105 and inhibitor IκBγ were both increased in AD brain tissue, indicating altered balance and feedback within the pathway.	Sample size and cellular source were NR in the abstract and direction cannot be inferred.	(Huang et al., 2005a)
(Pizzi, Sarnico et al. 2005)	Primary cortical neurons and neuronal cell lines challenged with Aβ.	mGlu5 agonists, c-Rel RNA interference or overexpression, and TAT-Bcl-xL rescue; concentrations and exposure durations: NR.	c-Rel knockdown vs overexpression and matched controls; TAT-Bcl-xL rescue vs no rescue.	Rodent in vitro	c-Rel was necessary and sufficient to induce MnSOD and Bcl-xL and protect neurons from Aβ toxicity. Providing Bcl-xL downstream restored survival when c-Rel signaling was impaired.	Protection was tested only in acute neuronal systems.	(Pizzi et al., 2005)
(Samuelsson, Fisher et al. 2005)	Mixed primary rat glial cultures.	Aβ25–35 and IL-1β alone or together over a concentration range; NF-κB DNA binding was measured at 30 min, 2 h, and 24 h. Exact concentrations: NR.	Aβ25–35 and interleukin−1β alone vs combined exposure and untreated controls across time.	Rodent in vitro	Aβ and interleukin−1β acted together to produce stronger and more persistent NF-κB DNA binding in primary glia than either stimulus alone.	Mixed glia prevent assignment to astrocytes versus microglia and no neuronal or disease outcome was measured.	(Samuelsson et al., 2005)
(Kim, Kim et al. 2003)	Human neuroblastoma cells exposed to Aβ42.	Aβ42 dose-response, PLC-δ1-promoter deletion/mutation, dominant-negative TAK1 or NIK, and NF-κB pathway manipulation; exact doses/times: NR.	Aβ42 dose conditions; wild-type vs deleted/mutated PLC-δ1 κB promoter; dominant-negative kinase conditions.	Human in vitro	Aβ42 activated the PLC-δ1 promoter through a functional κB site. Deleting or mutating this site prevented promoter activation.	The downstream relevance of PLC-δ1 induction to AD injury was not directly tested.	(Kim et al., 2003a)
(Rodriguez-Kern, Gegelashvili et al. 2003)	Differentiated primary rat astrocytes, with neuronal/glutamate-homeostasis relevance.	Subtoxic Aβ1–42 at 1–5 µM or BDNF; exposure duration and inhibitor doses: NR.	Aβ1–42 or BDNF exposure with pathway inhibitors vs untreated controls.	Rodent in vitro	Aβ and brain-derived neurotrophic factor increased astrocytic GLT−1/EAAT2 expression and glutamate uptake through NF-κB-dependent pathways.	Micromolar acute peptide and isolated astrocytes do not establish net protection in an amyloid-bearing brain.	(Rodriguez-Kern et al., 2003)
(Hattori, Arai et al. 2001)	postmortem AD and control brain tissue containing neurofibrillary pathology.	No exposure; Southwestern histochemistry and immunohistochemistry.	AD/disease vs control tissue.	Human ex vivo/biospecimen	Activated NF-κB and increased IκB were found together in neurofibrillary lesions, suggesting pathway activation accompanied by an attempted inhibitory feedback response.	Sample size was NR and static localization cannot identify cause or productive transcription.	(Hattori et al., 2001)
(Tomita, Fujita et al. 2000)	Neuronal cell systems expressing APP, RelA/p65, p50-containing dimers, and the neuron-specific adaptor X11L.	p65 or p50/dimer expression and X11L gain/loss or PDZ-domain disruption; transfection levels and durations: NR.	p65/p50 expression with or without X11L or PDZ-domain mutants vs matched controls.	In vitro	RelA/p65 selectively increased Aβ42 production. The neuronal adaptor X11L reduced this effect by binding p65 through its PDZ domain.	Subunit and binding-domain specificity support a direct mechanism, but overexpression may distort stoichiometry and no aged in-vivo validation was provided.	(Tomita et al., 2000a)
(Kaltschmidt, Uherek et al. 1999)	Primary neuronal cultures.	Preconditioning with 0.1 µM Aβ1–40 then 10 µM Aβ1–40 challenge; TNF-α produced maximal NF-κB activation at 2 ng/mL; dominant-negative IκBα tested pathway dependence.	0.1 µM Aβ preconditioning followed by 10 µM challenge vs non-preconditioned controls; dominant-negative IκBα conditions.	Rodent in vitro	Pre-exposure to low-dose Aβ1–40 protected neurons from a later high-dose challenge. Blocking NF-κB with dominant-negative IκBα removed this acquired resistance.	The 100-fold contrast establishes acute preconditioning but not chronic disease modification.	(Kaltschmidt et al., 1999a)
(Bales, Du et al. 1998)	Primary fetal-rat cortical neurons and primary rat astroglia exposed to Aβ.	Concentration- and time-dependent Aβ exposure with IκBα antisense manipulation; exact peptide concentrations: NR.	Aβ-exposed vs untreated neuronal and astroglial cultures; IκBα antisense vs control.	Rodent in vitro	During the same Aβ exposure, constitutive neuronal NF-κB declined as neuronal DNA fragmentation began, whereas astroglial NF-κB and inflammatory cytokines increased.	Fetal cells and acute peptide exposure differ substantially from aged multicellular AD.	(Bales et al., 1998)
(Guo, Robinson et al. 1998)	Differentiated PC12 cells expressing AD-linked PS1 L286V.	Secreted APPα or Aβ/stress challenges, κB-decoy blockade, oxidative stress, thapsigargin, and calcium perturbation; concentrations and durations: NR.	sAPPα rescue vs control in mutant-presenilin cells; κB DNA-decoy vs control decoy.	Rodent in vitro	Soluble APPα required NF-κB signaling to stabilize intracellular calcium and mitochondrial function and protect presenilin−1-mutant cells from apoptosis.	ΚB blockade supports mediation of sAPPα rescue, but PC12 cells and mutant-PS1 overexpression represent an acute familial-stress model.	(Guo et al., 1998a)
(Lukiw and Bazan 1998)	postmortem superior temporal neocortex from control and CERAD-classified sporadic AD cases; nuclear extracts from 20 cases, ages 60–82 years, postmortem interval 0.5–6.5 h.	No treatment; biochemical tissue comparison plus COX−2-promoter deletion constructs.	AD severity groups vs controls; intact vs deleted κB element in the COX−2 promoter.	Human ex vivo/biospecimen	NF-κB DNA binding was associated with κB-dependent COX−2 transcription in aging and sporadic AD neocortex.	Dimer-binding and promoter evidence strengthen biological plausibility, but cross-sectional bulk tissue remains vulnerable to cell-composition and postmortem effects.	(Lukiw and Bazan, 1998a)
(Hirsch, Agid et al. 1997)	Nucleus basalis of Meynert from four patients with AD and four controls, focusing on cholinergic neurons.	No treatment; cell-resolved postmortem immunohistochemistry.	AD vs control postmortem tissue.	Human observational/genetic; Human ex vivo/biospecimen	Nuclear NF-κB was more frequent in basal-forebrain cholinergic neurons from AD brains than controls.	Selective-vulnerability localization is valuable, but n = 4 per group and cross-sectional survivor bias preclude assigning activation as protective or pathogenic.	(Hirsch et al., 1997)
(Bonaiuto, McDonald et al. 1997)	N9 mouse microglia, primary rat microglia, and human monocytes.	Suboptimal Aβ25–35 combined with interferon-γ at 100 U/mL for at least 120 min; higher Aβ alone also activated cells. Exact suboptimal/high peptide concentrations: NR.	Aβ and interferon-γ alone vs combined exposure and untreated controls.	Human ex vivo/biospecimen; Rodent in vitro	Aβ and interferon-γ cooperated to activate RelA/p50 DNA binding in microglia and monocytes. Higher Aβ exposure could activate the pathway without cytokine costimulation.	Dimer and cross-species confirmation support inflammatory amplification, but transformed N9 cells and acute IFN-γ costimulation may exaggerate plaque-associated signaling.	(Bonaiuto et al., 1997a)
(Kaltschmidt, Uherek et al. 1997)	Primary neurons exposed to Aβ1–40 or Aβ25–35 and postmortem human neurons surrounding early plaques.	Maximal activation with 0.1 µM Aβ1–40 or 0.1 µM Aβ25–35; antioxidant blockade tested reactive-oxygen-intermediate dependence.	Aβ dose conditions with antioxidant or NF-κB inhibition vs controls; human plaque-adjacent vs control neurons.	Rodent in vitro; Human ex vivo	Low-concentration Aβ activated neuronal NF-κB through reactive oxygen intermediates. Activated p65 was also detected near early plaques in human brain tissue, but the functional outcome was not determined.	The experiment did not show whether activation itself improved or worsened neuronal outcome.	(Kaltschmidt et al., 1997a)
(Kitamura, Shimohama et al. 1997)	postmortem temporal cortex from AD and neurologically normal controls.	No treatment; subcellular fractionation and antibody-based protein analysis.	AD/disease vs control tissue.	Human ex vivo/biospecimen	AD temporal cortex showed redistribution and enrichment of p65 and STAT1 in particulate and nuclear fractions, consistent with an inflammatory transcriptional state.	Subcellular redistribution supports pathway engagement, but sample size and postmortem details were NR in the abstract and causality is unresolved.	(Kitamura et al., 1997a)
(Terai, Matsuo et al. 1996)	postmortem hippocampal formation, entorhinal and temporal cortex, and visual cortex from AD and control brains.	No treatment; regional p65 immunohistochemistry.	AD/disease vs control tissue.	Human ex vivo/biospecimen	p65 immunoreactivity was enriched in vulnerable hippocampal and cortical regions, neurofibrillary tangles, and dystrophic neurites in AD brain tissue.	Sample size was NR in the abstract and immunoreactivity cannot establish activation or direction.	(Terai et al., 1996a)

**Abbreviations**: AAV, adeno-associated virus; AD, Alzheimer disease; Aβ, amyloid-β; ALS, amyotrophic lateral sclerosis; APOE, apolipoprotein E; APP, amyloid precursor protein; BACE1, β-site amyloid precursor protein-cleaving enzyme 1; BBB, blood–brain barrier; BDNF, brain-derived neurotrophic factor; C3, complement component 3; C3aR, complement component 3a receptor; CERAD, Consortium to Establish a Registry for Alzheimer’s Disease; CFH, complement factor H; CHO, Chinese hamster ovary; CHOP, C/EBP homologous protein; COX−2, cyclooxygenase−2; CRISPR/Cas9, clustered regularly interspaced short palindromic repeats/CRISPR-associated protein 9; CypA, cyclophilin A; EAAT2, excitatory amino acid transporter 2; GSK3β, glycogen synthase kinase 3 beta; HDAC7, histone deacetylase 7; HEK293, human embryonic kidney 293; IFN-γ, interferon gamma; IκB, inhibitor of nuclear factor kappa B; IKK, inhibitor of nuclear factor kappa B kinase; IL-1β, interleukin−1 beta; iPSC, induced pluripotent stem cell; JNK, c-Jun N-terminal kinase; LOAD, late-onset Alzheimer disease; LPS, lipopolysaccharide; LRP1, low-density lipoprotein receptor-related protein 1; MCI, mild cognitive impairment; MMP9, matrix metalloproteinase 9; MnSOD, manganese superoxide dismutase; MSBB, Mount Sinai Brain Bank; NEMO, nuclear factor kappa B essential modulator; NF-κB, nuclear factor kappa B; NLRP3, NOD-like receptor family pyrin domain-containing 3; NMDA, N-methyl-D-aspartate; NR, not reported; PBMC, peripheral blood mononuclear cell; PDTC, pyrrolidine dithiocarbamate; PGE2, prostaglandin E2; PINK1, PTEN-induced kinase 1; PLC-δ1, phospholipase C delta 1; PRMT5, protein arginine methyltransferase 5; PS1, presenilin 1; PSP, progressive supranuclear palsy; RAGE, receptor for advanced glycation end products; RIPK1, receptor-interacting protein kinase 1; ROS, reactive oxygen species; SCA3, spinocerebellar ataxia type 3; SIRT1, sirtuin 1; sAPPα, soluble amyloid precursor protein alpha; TDP−43, TAR DNA-binding protein 43; TIGAR, TP53-induced glycolysis and apoptosis regulator; TLR, Toll-like receptor; TNF-α, tumor necrosis factor alpha; UCH-L1, ubiquitin C-terminal hydrolase L1.

## 3 Human Evidence for Context-Dependent NF-κB Dysregulation in AD

Human studies support a role for NF-κB signaling in AD, but they do not support a simple model in which greater pathway activity is always harmful and reduced activity is necessarily beneficial. Instead, the evidence points to biologically distinct forms of dysregulation. Impaired inducible NF-κB signaling may increase susceptibility to late-onset Alzheimer’s disease (LOAD), whereas persistent or exaggerated RelA/p65-associated activity is more commonly observed in MCI and established AD. These states are not contradictory: failure to mount an appropriate stress response differs fundamentally from prolonged NF-κB activation in cells already exposed to Aβ, tau pathology, or chronic inflammation.

The clearest human evidence for impaired inducible signaling comes from studies of SHANK-associated RH domain-interacting protein (SHARPIN), a component of the linear ubiquitin chain assembly complex (LUBAC). Whole-exome sequencing of 202 APOE ε4-negative individuals with LOAD identified the rare SHARPIN G186R variant, which was subsequently associated with markedly increased LOAD risk in 4563 cases and 16,459 controls (OR: 6.1). Functionally, cells carrying G186R showed reduced NF-κB responsiveness after stimulation (Asanomi et al., 2019b). A second SHARPIN variant, R274W, was identified through whole-genome sequencing of 180 individuals with LOAD and 184 with MCI and was subsequently tested in 5043 LOAD cases and 11,984 controls. Although its association with LOAD was more modest (OR: 1.43), R274W similarly altered SHARPIN localization and reduced stimulus-induced NF-κB signaling (Asanomi et al., 2022b). The convergence of these findings suggests that intact SHARPIN–LUBAC function may be important for mounting an appropriate NF-κB response to cellular stress.

Experimental introduction of G186R using CRISPR/Cas9 provided a further link to Aβ biology. Homozygous G186R introduced into HEK293 cells produced weaker TNF-α-induced NF-κB signaling together with increased extracellular Aβ40 and Aβ42 secretion (Asanomi et al., 2024). This supports a biological connection among SHARPIN dysfunction, impaired inducible NF-κB signaling, and altered Aβ metabolism, but it does not establish that reduced NF-κB activity directly caused the increase in Aβ. SHARPIN may influence APP processing, trafficking, degradation, or secretion through additional mechanisms, and the use of a rare homozygous clone in a non-neural cell line limits extrapolation to heterozygous human carriers. The SHARPIN findings therefore argue that loss of normally regulated NF-κB responsiveness may increase disease vulnerability, not that generalized NF-κB activation would be protective.

Common NF-κB-related genetic variation is more difficult to interpret mechanistically. In a Spanish case-control study of 639 individuals with LOAD and 500 cognitively healthy controls, the CC genotype of NFKB1 rs7667496 was associated with LOAD, whereas the examined NFKBIA and NFKBIZ variants were not (Vazquez-Coto et al., 2025b). Because NFKB1 encodes p105 and its processed product p50, which can participate either in transcriptionally active p50/RelA complexes or in repressive p50/p50 homodimers, the genetic association alone does not indicate whether disease risk reflects increased activation, reduced repression, altered p105 processing, or another linked mechanism (Savinova et al., 2009, Somma et al., 2021).

Postmortem studies provide a different view by demonstrating NF-κB abnormalities directly in vulnerable human brain regions. Early investigations reported enhanced or altered RelA/p65 immunoreactivity in hippocampal and cortical regions, neurofibrillary tangles, dystrophic neurites, cholinergic neurons of the nucleus basalis of Meynert, and temporal cortex (Hirsch et al., 1997, Kitamura et al., 1997b, Terai et al., 1996b). These observations established that NF-κB components are altered in regions affected by AD, but localization or nuclear accumulation of p65 alone cannot determine whether the pathway was driving inflammatory transcription, participating in a compensatory stress response, or accumulating secondary to cellular injury.

Stronger functional evidence came from studies linking NF-κB DNA binding to specific transcriptional outputs. Lukiw et al. found that NF-κB DNA-binding activity in superior temporal neocortex closely paralleled transcription from the κB-responsive COX−2 promoter in sporadic AD brain (Lukiw and Bazan, 1998b). This finding is consistent with a pro-inflammatory NF-κB program in established disease, although the cross-sectional postmortem design cannot determine whether pathway activation preceded or followed neurodegeneration. Other studies further showed that NF-κB dysregulation cannot be described simply as uniform pathway activation. Increased IκB expression was observed alongside NF-κB activation and neurofibrillary pathology, while p105 and IκBγ were also elevated in AD brain (Huang et al., 2005b, Yoshiyama et al., 2001). Because these proteins have inhibitory or regulatory functions, their accumulation may reflect compensatory negative feedback, altered processing or degradation, or coexistence of different signaling states across cell types and brain regions.

NF-κB abnormalities are also associated with tau-related pathology. Ohta et al. identified a nuclear complex containing TDP−43 and p65 in temporal cortex, with the most prominent signal observed in four individuals with MCI and episodic-memory impairment (Ohta et al., 2014). Yamaguchi et al. later detected phosphorylated p65 within granulovacuolar degeneration structures and tau-positive neurites, particularly in AD tissue (Yamaguchi et al., 2019b). Together, these studies support a spatial association between altered NF-κB signaling and tau-related neuronal pathology across the MCI–AD spectrum. However, they do not establish that NF-κB initiates tau aggregation or drives its propagation, because phosphorylated or sequestered p65 within pathological structures could also reflect impaired clearance or a response to existing neuronal injury.

Altered NF-κB signaling in AD is not restricted to the brain. Ascolani et al. found that mitogen-stimulated peripheral blood mononuclear cells from individuals with AD showed increased NFKB1 expression, greater inducible p50/p65 DNA binding, and altered NF-κB target-gene expression, with some measures related to cognitive severity (Ascolani et al., 2012). These findings indicate systemic immune dysregulation, although peripheral NF-κB activity should not be assumed to mirror signaling within the brain. Cholinergic dysfunction may provide another regulatory influence. In AD brain tissue and Aβ1–42-treated neuronal and glial cell models, reduced α3 nicotinic acetylcholine receptor expression was associated with higher p65, CCL2, and CCL3 levels, while α3 nAChR knockdown reproduced part of the inflammatory response (Liao et al., 2016). This suggests that loss of cholinergic restraint may contribute to NF-κB-associated inflammation.

Human induced pluripotent stem cell (iPSC) models have provided more controlled evidence for cell-specific effects of AD risk factors on NF-κB signaling. Arnaud et al. compared isogenic APOE3/3, APOE4/4, and APOE-null iPSC-derived astrocytes and found that APOE4 reduced transgelin 3 (TAGLN3) expression and increased NF-κB responsiveness. Restoring TAGLN3 or pharmacologically modifying the pathway attenuated the inflammatory phenotype (Arnaud et al., 2022b). These findings suggest that APOE4 can shift astrocytes toward a state that is more responsive to NF-κB-mediated inflammatory signaling.

A related mechanism links astrocytic lipid handling to NF-κB activity. Hamilton et al. found increased fatty acid-binding protein 7 (FABP7) in plaque-associated astrocytes across AD-related mouse models, human AD tissue, and human iPSC-derived astrocytes. Overexpression of ligand-binding-competent FABP7 induced a broad NF-κB-associated inflammatory transcriptional program, whereas a ligand-binding-deficient mutant did not (Hamilton et al., 2024b). This supports a mechanistic connection between altered lipid handling and inflammatory NF-κB signaling in astrocytes. However, both APOE4- and FABP7-based models rely partly on isolated or reprogrammed astrocytes, which incompletely reproduce aging and the interactions with neurons, microglia, amyloid, tau, and the neurovasculature present in the human AD brain.

Taken together, the human evidence is most coherent when NF-κB is viewed as a dynamic, context-dependent signaling system rather than as a pathway that is uniformly increased or suppressed throughout AD. SHARPIN genetics suggests that impaired inducible NF-κB responsiveness may increase susceptibility to LOAD, whereas postmortem and human-cell studies more often identify lesion-associated, inflammatory, or exaggerated RelA/p65 signaling once disease pathology is established. APOE4, FABP7, cholinergic dysfunction, and peripheral immune changes further indicate that genetic, metabolic, and systemic factors can modify the threshold and character of NF-κB responses.

The apparent duality of NF-κB therefore reflects different failures of pathway regulation rather than a true contradiction. Genetic studies can identify abnormalities that may precede clinical disease but often cannot specify the responsible cell type or downstream mechanism; postmortem studies reveal pathway abnormalities in vulnerable brain regions but cannot establish temporal causality; peripheral studies demonstrate systemic immune changes but do not directly represent brain signaling; and isogenic human-cell models enable mechanistic investigation but incompletely reproduce aging and multicellular interactions. The available human evidence therefore supports neither global NF-κB activation nor global inhibition. Instead, preservation of appropriately regulated NF-κB responsiveness may support cellular homeostasis, whereas persistent, cell-specific, and RelA/p65-biased signaling appears increasingly associated with inflammatory and neurodegenerative pathology across the symptomatic AD spectrum.

## 4 Neuronal NF-κB Signaling: From Adaptive Stress Responses to Pathological Signaling

Evidence from neuronal models helps explain why NF-κB has been assigned apparently opposing roles in AD. The discrepancy becomes less paradoxical when the nature of the signaling response is considered. Brief and tightly regulated NF-κB activation can participate in neuronal stress adaptation, trophic signaling, antioxidant defense, calcium homeostasis, and non-amyloidogenic processing of APP. By contrast, persistent or disease-conditioned signaling, particularly through IKKβ and RelA/p65, is more consistently associated with BACE1 expression, Aβ production, mitochondrial dysfunction, impaired protein degradation, inflammatory signaling, and regulated cell death. Thus, the biological consequence of neuronal NF-κB activity depends on the initiating stimulus, duration and intensity of activation, NF-κB dimer, promoter and cofactor environment, and downstream genes engaged.

Some of the strongest evidence for a protective neuronal response comes from low-intensity Aβ exposure. Kaltschmidt et al. showed that relatively low concentrations of Aβ1–40 or Aβ25–35, with maximal responses around 0.1 µM, activated NF-κB in primary neurons through reactive oxygen intermediates; activated p65 was also observed in neurons near early plaques in human AD tissue (Kaltschmidt et al., 1997b). A subsequent preconditioning study demonstrated the functional significance of this response: neurons initially exposed to 0.1 µM Aβ1–40 became more resistant to a later 10 µM challenge, whereas blocking NF-κB with dominant-negative IκBα abolished the protection and increased susceptibility to Aβ toxicity (Kaltschmidt et al., 1999b). TNF-α similarly produced an NF-κB-dependent protective response. These findings support a model in which mild stress can recruit an adaptive neuronal NF-κB program, but they do not imply that Aβ itself is beneficial or that prolonged NF-κB activation would protect the chronically diseased brain.

Other trophic pathways point in the same direction. In differentiated PC12 cells carrying the familial AD-associated presenilin−1 L286V mutation, soluble APPα (sAPPα) protected against oxidative stress, calcium dysregulation, mitochondrial dysfunction, and apoptosis, whereas blockade of NF-κB-dependent transcription abolished this protection (Guo et al., 1998b). Similarly, erythropoietin reduced Aβ-induced apoptotic injury in primary hippocampal neurons, but the effect was substantially weakened by p65 knockdown or inhibition of its nuclear translocation (Chong et al., 2005). These studies show that RelA/p65 is not intrinsically neurotoxic; when transiently recruited downstream of trophic signals, it can participate in neuronal survival.

A more clearly defined protective transcriptional program involves c-Rel. In Aβ-challenged cortical neurons and neuronal cell lines, stimulation of metabotropic glutamate receptor 5 induced c-Rel-dependent expression of manganese superoxide dismutase (MnSOD) and Bcl-xL, supporting antioxidant and anti-apoptotic protection (Pizzi et al., 2005). Gain- and loss-of-function experiments established that c-Rel was both necessary and sufficient for this response, while TAT–Bcl-xL restored survival when c-Rel signaling was impaired. This provides one of the clearest examples of a specific protective NF-κB transcriptional program, although the mechanism was demonstrated mainly under acute experimental conditions.

Calcium-dependent NF-κB signaling may also support neuronal adaptation. In neurons expressing wild-type or AD-associated mutant presenilin−1, endoplasmic-reticulum calcium release activated NF-κB and suppressed Aβ-associated expression of C/EBP homologous protein (CHOP), a mediator of apoptosis during unresolved ER stress (Schapansky et al., 2007b, Choi et al., 2006b). In M3 muscarinic receptor-expressing SH-SY5Y cells, oxotremorine-M similarly promoted calcium entry, p65 nuclear translocation, and sAPPα release, whereas inhibition of calcium entry or NF-κB reduced the response (Choi et al., 2006b). Because α-secretase cleavage generates sAPPα and prevents the same APP molecule from entering the amyloidogenic pathway, these findings suggest that under specific conditions NF-κB can favor non-amyloidogenic APP processing. Overall, however, these protective effects were observed during brief, stimulus-specific signaling over hours or days and therefore do not establish sustained canonical NF-κB activation as beneficial in chronic AD.

The direction of signaling changes when NF-κB becomes coupled to amyloidogenic APP processing. Tomita et al. showed that RelA/p65 overexpression selectively increased Aβ42 secretion, whereas the neuronal adaptor X11-like protein restrained this effect through a PDZ-domain-dependent interaction with p65 (Tomita et al., 2000b). More compelling mechanistic evidence emerged from studies of the BACE1 promoter. Bourne et al. found that NF-κB-associated regulation could restrain BACE1 promoter activity in resting neurons, whereas Aβ exposure in neurons and activation of astrocytes shifted the same κB-responsive region toward increased BACE1 transcription (Bourne et al., 2007b). Chen et al. subsequently demonstrated that TNF-α-induced BACE1 transcription required both RelA and an intact κB-binding sequence; mutation of the κB site or removal of RelA reduced BACE1 expression, APP processing, and Aβ generation (Chen et al., 2012b). These findings show that the same pathway can exert different transcriptional effects depending on cellular state and inflammatory context.

A dose-dependent shift may help explain this transition. Chami et al. reported that lower Aβ exposure was associated with suppression of βAPP and amyloidogenic secretase expression, whereas higher, supraphysiological Aβ concentrations reversed the response and increased βAPP and β- and γ-secretase components (Chami et al., 2012b). Although the use of HEK293 cells limits direct extrapolation to neurons, the bidirectional response supports the possibility that increasing Aβ-associated stress can shift NF-κB from a compensatory to an amyloidogenic state.

Once established, this state can reinforce BACE1 through more than one mechanism. Bcl−2-associated athanogene 1 M (BAG-1M) acted as a coactivator of NF-κB-dependent BACE1 transcription, increasing BACE1 expression and Aβ production, while hippocampal BAG-1M overexpression increased amyloid pathology and memory impairment in an AD mouse model (Shi et al., 2017). In parallel, Aβ1–42-induced NF-κB activation reduced ubiquitin C-terminal hydrolase L1 (UCH-L1) in neuronal cells, impairing cathepsin D-dependent lysosomal degradation of BACE1 and allowing the enzyme to accumulate (Guglielmotto et al., 2012b). Together, these studies show that disease-conditioned NF-κB signaling can increase BACE1 both by promoting its transcription and by limiting its degradation. Pharmacological studies are consistent with this interpretation: NF-κB inhibitors reduced Aβ40 and Aβ42 production in APP-expressing cells, whereas CD40 ligation increased APP metabolites and Aβ generation through partially NF-κB-dependent signaling (Ait-Ghezala et al., 2007b, Paris et al., 2007b), although these approaches are less mechanistically specific.

NF-κB-dependent neuronal transcription also extends beyond BACE1. Aβ42 increased phospholipase C delta 1 (PLC-δ1) expression through a functional κB element in its promoter, and mutation or deletion of this element abolished the response (Kim et al., 2003b). Similarly, experiments in primary rat cerebellar cultures exposed to 1–2 µM Aβ1–40 for 6–24 h showed that the strongest response occurred after approximately 12 h at 1 µM and involved NMDA-receptor-sensitive formation of both p50/RelA and p50/p50 complexes (Kawamoto et al., 2008b). The coexistence of transcriptionally distinct dimers reinforces the point that “NF-κB activation” does not represent a single molecular state, and that dimer composition can influence the resulting transcriptional program.

Mitochondrial dysfunction provides another link between pathological NF-κB signaling and neuronal injury. In HT22 hippocampal cells and isolated mitochondria, Aβ activated an intramitochondrial IκBα/NF-κB pathway associated with reduced cytochrome-c oxidase subunit III expression, impaired respiratory activity, and neuronal injury (Shi et al., 2014b). A complementary mechanism was identified through PTEN-induced kinase 1 (PINK1), a regulator of mitochondrial quality control. Loss or knockdown of PINK1 in Aβ-producing neuronal systems and AD-model mice increased mitochondrial reactive oxygen species, phosphorylation of NFKB1/p50 and RelA/p65, BACE1 and γ-secretase activity, and Aβ accumulation. Conversely, increasing neuronal PINK1 suppressed Aβ-induced NF-κB activation and amyloidogenic processing, while scavenging mitochondrial reactive oxygen species similarly reduced NF-κB activity, normalized APP processing, and limited Aβ accumulation (Du et al., 2025). These findings support a PINK1–mitochondrial ROS–NF-κB–APP-processing axis linking defective mitochondrial quality control to amyloidogenic signaling.

Persistent neuronal NF-κB activity can also promote inflammatory cell-death pathways. Lei et al. found increased NF-κB and miR−146a−5p together with reduced TP53-induced glycolysis and apoptosis regulator (TIGAR) in AD serum samples and Aβ25–35-exposed human hippocampal neurons (Lei et al., 2021). In the neuronal model, miR−146a−5p directly suppressed TIGAR, while NF-κB knockdown or restoration of TIGAR reduced oxidative stress and pyroptosis; reintroduction of miR−146a−5p weakened these protective effects. Because TIGAR supports pentose-phosphate pathway activity and NADPH-dependent antioxidant defense, these findings provide a plausible mechanism linking NF-κB-dependent microRNA signaling to impaired redox control and inflammatory neuronal death. A related pathway was identified by Zhang et al., who found increased TLR2 in individuals with amnestic MCI and AD and examined its function in Aβ1–42-treated SH-SY5Y cells and APP/PS1 mice. TLR2 suppression reduced NF-κB/NLRP3 signaling, pyroptosis, neuronal injury, AD-related pathology, and behavioral impairment, whereas NF-κB overexpression or direct NLRP3 activation partially reversed these benefits (Zhang et al., 2026b). These reversal experiments provide stronger support for a TLR2 → NF-κB → NLRP3 → pyroptosis pathway than would parallel changes in pathway markers alone. Nevertheless, both mechanisms remain supported primarily by experimental models and do not establish that neuronal pyroptosis driven by these pathways is a dominant process in sporadic human AD.

Broader in-vivo evidence nevertheless supports a detrimental role for persistent neuronal signaling in established disease. Schnöder et al. conditionally deleted Ikbkb, encoding IKKβ, specifically in neurons of APP- and tau-transgenic mice and complemented these experiments with neuronal cell models. Neuronal IKKβ deficiency reduced BACE1 expression and Aβ accumulation as well as phosphorylated tau, apoptosis, and inflammatory abnormalities (Schnöder et al., 2023). These findings place chronic neuronal IKKβ activity upstream of several major AD-related pathological processes and provide stronger causal evidence than studies based solely on pathway activation markers. Cognitive and synaptic improvement, however, was not uniform across the transgenic models, indicating that reducing molecular pathology may not be sufficient to restore neural networks once substantial damage has occurred. Moreover, because IKKβ acts on substrates beyond canonical NF-κB signaling, not every consequence of Ikbkb deletion can be attributed specifically to RelA-dependent transcription.

Taken together, the neuronal literature is better understood as evidence for distinct NF-κB signaling states rather than a simple protective-versus-harmful paradox. Brief activation during mild stress or trophic stimulation can support preconditioning, calcium homeostasis, non-amyloidogenic APP processing, antioxidant defense, and resistance to apoptosis, with the c-Rel–MnSOD/Bcl-xL pathway providing the clearest defined protective transcriptional program. In contrast, persistent signaling in an Aβ-rich, inflammatory, oxidatively stressed, or metabolically compromised environment increasingly engages IKKβ/RelA-associated BACE1 transcription, impaired BACE1 degradation, amyloidogenic processing, mitochondrial dysfunction, inflammatory microRNA signaling, NLRP3 activation, and pyroptotic cell death. The distinction is therefore not simply low versus high NF-κB activity, nor can it be reduced entirely to early versus late disease. The biological outcome depends on the signaling configuration and cellular context. Protective experiments explain why complete suppression of neuronal NF-κB could remove important physiological stress responses, whereas promoter-level studies, rescue and reversal experiments, and neuron-specific IKKβ deletion provide stronger causal evidence that sustained, disease-conditioned IKKβ/RelA signaling represents the more consistently pathogenic form of neuronal NF-κB activity in AD.

## 5 Microglial NF-κB Signaling: Distinguishing Amyloid Clearance, Inflammation, and Tau Propagation

The role of NF-κB in microglia cannot be reduced to a simple balance between beneficial amyloid clearance and harmful inflammation. Microglia perform multiple functions in AD, including migration toward pathological deposits, uptake and degradation of Aβ and tau, cytokine production, synaptic regulation, trophic support, and host defence. These functions are related but distinct. In particular, increased uptake of Aβ or tau does not necessarily indicate complete intracellular degradation, and reduced plaque or aggregate burden does not by itself demonstrate reduced neurotoxicity. Across experimental studies, several microglial surveillance and cargo-uptake functions remain intact when sustained IKK–NF-κB inflammatory signaling is suppressed, whereas persistent pathway activity is more consistently associated with inflammatory neuronal injury and, in tauopathy models, propagation of seeding-competent tau.

Studies targeting PARP1 and IKKβ illustrate this distinction particularly well. In hAPP-J20 mice and complementary microglial models, PARP1 deficiency reduced Aβ-induced NF-κB activation, nitric oxide and TNF-α production, microglial neurotoxicity, and cognitive abnormalities without impairing Aβ phagocytosis; trophic factors including TGF-β and VEGF were also increased (Kauppinen et al., 2011b). Similarly, conditional deletion of Ikbkb in the myeloid lineage of TgCRND8 mice reduced TNF-α and IL-1β, cerebral Aβ accumulation, and inflammatory activation while preserving synaptic proteins and improving memory performance (Liu et al., 2014). Microglial recruitment to plaques remained intact, and IKKβ-deficient macrophages showed enhanced uptake of oligomeric Aβ42. Together, these findings indicate that chronic canonical NF-κB signaling is not required for microglial recognition or internalization of Aβ. They do not, however, establish that all subsequent steps of lysosomal degradation remain unaffected.

The importance of separating phagocytosis from inflammatory state is further demonstrated by Nfkb1 deletion. Rolova et al. found that loss of p50 increased microglial Aβ uptake and modestly reduced amyloid burden, but simultaneously increased inflammatory gene expression and leukocyte infiltration (Rolova et al., 2014). This apparently conflicting phenotype is consistent with the dual regulatory role of p50, which can participate in transcriptionally active p50/RelA complexes but can also form p50/p50 homodimers that restrain inflammatory transcription. Thus, greater Aβ uptake can coexist with a more inflammatory microglial state, showing that neither phagocytosis nor plaque burden alone is sufficient to define whether a microglial response is beneficial.

Several studies further indicate that the inflammatory environment can amplify and prolong microglial NF-κB signaling. Bonaiuto et al. showed that interferon-γ markedly enhanced RelA/p50 activation produced by relatively weak Aβ25–35 stimulation in microglia and human monocytes (Bonaiuto et al., 1997b). Likewise, Aβ25–35 and IL-1β produced a stronger and more sustained NF-κB response when combined than when applied individually (Samuelsson et al., 2005). These findings suggest that prior inflammatory activation can lower the threshold for subsequent Aβ-induced NF-κB signaling and potentially promote a persistent inflammatory state.

A direct functional connection between microglial NF-κB and neuronal injury was subsequently provided by Chen et al. In Aβ-stimulated microglia–neuron systems, constitutive inhibition of microglial NF-κB using a nondegradable IκBα super-repressor markedly reduced Aβ-dependent neuronal toxicity. Aβ also increased acetylation of RelA/p65 at lysine 310, a post-translational modification that enhances the transcriptional competence of RelA. Increasing sirtuin 1 (SIRT1), which deacetylates RelA, or treating the cells with the SIRT1-activating compound resveratrol reduced NF-κB signaling and protected neighboring neurons (Chen et al., 2005b). The convergence of direct pathway blockade and regulation of RelA acetylation strengthens the causal interpretation. In this system, microglial NF-κB did not merely accompany neuronal injury; suppression of the microglial pathway reduced that injury. The experiment therefore identifies an important non-cell-autonomous mechanism in which Aβ activates RelA-dependent transcription in microglia, and the resulting microglial signals subsequently damage neurons.

Not all microglial NF-κB activity is initiated directly by Aβ. Receptor-proximal regulatory mechanisms can determine how readily microglia enter an inflammatory state. Low-density lipoprotein receptor-related protein 1 (LRP1), which participates in ligand uptake as well as intracellular signaling, appears to function as one such restraint. In primary mouse microglia, Lrp1 knockdown or pharmacological blockade with receptor-associated protein increased c-Jun N-terminal kinase (JNK) and NF-κB signaling and exaggerated inflammatory cytokine production, particularly following lipopolysaccharide stimulation. Conversely, NF-κB inhibition reduced cytokine production and partially restored LRP1 expression suppressed by inflammatory stimulation (Yang et al., 2016). These findings suggest that LRP1 normally restrains microglial inflammatory responsiveness and that loss of this restraint can facilitate NF-κB activation. Because LRP1 also participates in ligand-clearance biology, its dysfunction provides a plausible point at which impaired handling of extracellular material and excessive inflammation could converge. Nevertheless, the experiment did not directly test Aβ or tau pathology in an AD model, so it identifies a regulatory mechanism relevant to AD rather than demonstrating that LRP1–NF-κB signaling drives disease progression.

MicroRNA regulation provides another mechanism through which transient inflammatory activation may become more persistent. Zhao et al. identified miR−25802 as a microRNA increased in the plasma of individuals with AD and in the hippocampus of APP/PS1 and 5xFAD mice. Using gain- and loss-of-function experiments in EOC20 microglial cells and AD mouse models, they showed that miR−25802 targets Krüppel-like factor 4 (KLF4), a transcription factor associated in this context with a less inflammatory microglial state. Increasing miR−25802 reduced KLF4 and enhanced NF-κB-associated inflammatory signaling, whereas restoring KLF4 attenuated microglial inflammation and improved pathological and cognitive outcomes (Zhao et al., 2024b). The rescue experiment is important because it orders the pathway more convincingly than parallel expression changes alone: the findings support a miR−25802 → KLF4 suppression → NF-κB activation sequence. Nevertheless, the mechanistic work relied partly on an immortalized microglial line and familial amyloid models, so whether the same regulatory circuit dominates in aged sporadic human AD remains unknown.

A related microRNA study identified a mechanism capable of stabilizing IKKβ itself. Wang et al. found that miR−155–5p suppresses S-phase kinase-associated protein 2 (SKP2), thereby reducing ubiquitination and degradation of IKKβ. As a result, IKKβ protein becomes more stable and remains available for continued signaling. In APP/PS1 mice and Aβ1–42-treated N2a cells, inhibition of miR−155–5p or knockdown of IKKβ reduced Aβ deposition and improved pathological and cognitive outcomes, whereas loss of SKP2 produced the opposite phenotype (Wang et al., 2022a). This study provides a plausible mechanism by which inflammatory kinase activity can become persistent: rather than merely increasing acute IKKβ activation, miR−155–5p reduces the cellular machinery responsible for IKKβ turnover. However, this experiment was not microglia-specific; the cellular work used N2a neuronal cells and the in-vivo manipulations were performed in the hippocampus. It should therefore be regarded as supporting evidence for an AD-relevant miR−155–SKP2–IKKβ mechanism rather than definitive evidence that the same sequence operates specifically within microglia.

More recent work has identified a receptor–adaptor mechanism that does include microglial inflammatory signaling. Liu et al. integrated human AD transcriptomic datasets with Aβ1–42-treated SH-SY5Y cells, LPS-stimulated HMC3 microglia, and APP/PS1 mice to examine signaling lymphocytic activation molecule family member 8 (SLAMF8) and nerve injury-induced protein 2 (NINJ2). SLAMF8 was increased in the disease models, and its overexpression enhanced TLR4/NF-κB signaling, pro-inflammatory cytokine production, oxidative stress, and AD-like abnormalities. NINJ2 interacted functionally with SLAMF8, and NINJ2 loss prevented SLAMF8-induced p65 activation and markedly attenuated the inflammatory and oxidative phenotype (Liu et al., 2025). This pathway-ordering experiment supports a SLAMF8–NINJ2–TLR4/NF-κB axis more strongly than simple pathway colocalization. However, because different experimental stimuli were applied to neuronal and immortalized microglial lines, the relative contribution of this mechanism within specific cell populations of the aged sporadic AD brain remains unresolved.

The strongest evidence for a detrimental microglial NF-κB program comes from studies of tau, where the relevant outcome is not simply aggregate uptake but the release and intercellular propagation of seeding-competent tau. Wang et al. showed that primary mouse microglia exposed for 24 h to full-length tau fibrils at 2 µg/mL or K18/PL tau fibrils at 2.5 µg/mL strongly activated an NF-κB reporter, with the response approaching that produced by 50 ng/mL lipopolysaccharide. To examine what microglia subsequently did with pathological tau, the investigators exposed them to AD-derived tau at 1 µg/mL after two hours of pretreatment with 0.1 µM TPCA−1, an IKKβ inhibitor. IKKβ inhibition reduced the release of tau capable of seeding new aggregates and improved chaperone-mediated autophagy (Wang et al., 2022a). Thus, inhibition did not merely reduce an inflammatory marker; it changed the biological fate of tau processed by microglia.

The in-vivo component of the same study substantially strengthened this conclusion. Tamoxifen-inducible Cx3cr1-CreERT2 was used to either delete or constitutively activate microglial Ikbkb in PS19 tauopathy mice. In the tau-seeding experiments, three-month-old animals received unilateral hippocampal K18/PL tau inoculation—2 µL at 2.5 µg/µL in the loss-of-function experiments and 2 µL at 0.2 µg/µL in the gain-of-function experiments—and were examined one month later. Chronic outcomes were also assessed in 8–10-month-old PS19 mice using histopathology, Morris water-maze testing, bulk RNA sequencing, and single-nucleus RNA sequencing. Microglial IKKβ/NF-κB inactivation reduced tau seeding and anatomical spread, improved autophagic function, shifted disease-associated microglial transcription toward a less pathological state, and improved cognitive performance. Constitutive microglial IKKβ activation produced the opposite result and intensified tau propagation (Wang et al., 2022a). The bidirectional, microglia-specific genetic design provides unusually strong evidence that IKKβ–NF-κB activity is not merely associated with tau pathology but actively facilitates its intercellular propagation in this model.

An apparently paradoxical result from these experiments further illustrates why pathological endpoints must be considered separately. Chronic microglial NF-κB inhibition increased the amount of tau remaining within neuronal inclusions even though extracellular seeding, anatomical propagation, microgliosis, and cognitive impairment were reduced. This finding initially appears to indicate worsening pathology, but it is compatible with reduced processing and extracellular transfer of seeding-competent tau: more pathological tau remained within affected neurons, while less was propagated to new cells. The experiment therefore separates four outcomes that are often treated as interchangeable—intracellular tau burden, extracellular seeding activity, anatomical spread, and neurotoxicity. It does not demonstrate that long-term intracellular tau retention is harmless, but it strongly cautions against judging therapeutic benefit solely by the amount of intracellular aggregate detected.

The therapeutic relevance of the NF-κB inflammatory axis is also supported by a broader intervention study targeting NF-κB together with the NOD-like receptor protein 3 (NLRP3) inflammasome. Wahl et al. administered a brain-targeted nanoligomer cocktail directed against both NF-κB and NLRP3 for four weeks to 19-month-old wild-type mice and young rTg4510 tauopathy mice. Treatment reduced brain inflammatory cytokines and glial activation, improved cognitive performance, and favorably altered tau-related pathology and transcriptomic signatures associated with inflammation and neuronal health (Wahl et al., 2024). These findings support the therapeutic relevance of the broader NF-κB–NLRP3 inflammatory network in aging and tauopathy. However, because NF-κB and NLRP3 were targeted simultaneously and the intervention was not restricted specifically to microglia, the study cannot determine how much of the benefit resulted from NF-κB inhibition alone or from a particular microglial NF-κB program.

Taken together, the microglial evidence provides little support for the idea that sustained NF-κB activation is necessary for beneficial clearance functions. PARP1 deficiency and myeloid IKKβ deletion show that inflammatory and neurotoxic signaling can be reduced while microglial recruitment and Aβ uptake remain preserved, whereas p50 deficiency demonstrates that enhanced Aβ uptake can coexist with greater inflammation. Studies of inflammatory priming, RelA acetylation, LRP1, microRNAs, and receptor–adaptor pathways further explain how inducible NF-κB activity can become amplified or persistently maintained. Most importantly, microglia-specific gain- and loss-of-function experiments demonstrate that sustained IKKβ–NF-κB signaling can alter tau processing and actively promote the release and propagation of seeding-competent tau.

The apparent duality of microglial NF-κB therefore arises largely because NF-κB regulates only part of a much broader microglial functional repertoire. Suppressing pathological NF-κB signaling is not equivalent to suppressing microglial function as a whole. Microglia remain essential for surveillance, host defence, debris recognition, appropriate phagocytosis, lysosomal processing, trophic support, and synaptic homeostasis. A more plausible therapeutic strategy would therefore be to restrain persistent IKK–RelA-dependent inflammatory and tau-propagating programs while preserving physiological microglial functions, including recruitment, appropriate cargo uptake, and effective intracellular degradation.

## 6 Astrocytic NF-κB Signaling: Compensatory Functions and Neurotoxic Reactivity

Astrocytic NF-κB signaling illustrates particularly well why this pathway cannot be classified as uniformly protective or harmful in AD. Astrocytes regulate extracellular glutamate, participate in Aβ handling and plaque organization, communicate with microglia, contribute to extracellular protein degradation, and release mediators that influence neuronal function. NF-κB affects these processes differently. In some settings, astrocytic NF-κB appears to support compensatory responses to established pathology; in others, it suppresses Aβ degradation or drives inflammatory and neurotoxic signaling. Consequently, changes in plaque burden, astrogliosis, or inflammatory markers alone are insufficient to determine whether an astrocytic response is beneficial.

Early studies already suggested that reducing astrocytic or glial inflammation does not necessarily improve amyloid pathology. In APPswe/PS1dE9 mice, systemic treatment with the NF-κB inhibitor pyrrolidine dithiocarbamate reduced cyclooxygenase−2 (COX−2), tumor necrosis factor-α (TNF-α), and astrogliosis but increased cerebral Aβ42 (Zhang et al., 2009). Although the intervention was not astrocyte specific and PDTC has additional antioxidant and metal-chelating effects, the finding suggested that glial inflammatory responses may also contain compensatory functions relevant to Aβ handling.

More direct evidence for a compensatory astrocytic response came from selective activation of IKK2/NF-κB in astrocytes of APP23 mice. As expected, astrocytic pathway activation produced marked astrogliosis and induced prominent microglial reactivity. Surprisingly, however, both the number and size of Congo-red-positive amyloid plaques decreased in the cortex and hippocampus. Nearby microglia also acquired a transcriptional profile compatible with increased plaque-associated Aβ handling, whereas direct IKK2/NF-κB activation in microglia produced a more conventionally inflammatory phenotype (Yang et al., 2021b). These findings suggest that astrocytic NF-κB can modify amyloid pathology indirectly by changing astrocyte–microglia communication and possibly neuronal APP processing.

The reduction in compact plaques, however, should not be equated with global neuroprotection. Congo-red staining predominantly detects fibrillar amyloid deposits and does not describe the abundance of soluble oligomeric Aβ species, which can exert substantial synaptic toxicity. Nor did the study demonstrate comparable improvements across neuronal survival, synaptic physiology, and cognition. The supported interpretation is therefore narrower: astrocytic NF-κB can organize a plaque-associated glial response that alters deposited amyloid, but the experiment does not establish that chronic pathway activation improves overall brain function.

A later study provided particularly useful evidence that the effect of astrocytic NF-κB depends on the disease context. Huat et al. combined human AD transcriptomic and postmortem analyses with selective astrocytic NF-κB manipulation in mice. In otherwise healthy mice, sustained activation of astrocytic NF-κB disrupted the brain proteomic environment, reducing mitochondrial-associated proteins while increasing inflammatory proteins. Chronic activation also induced microglial reactivity and was associated with accumulation of the senescence-related protein p16^INK4A^ in neurons. Thus, persistent astrocytic NF-κB activation imposed on an otherwise healthy brain was clearly not homeostatic (Jong Huat et al., 2024). The result was different when NF-κB was inhibited in an established AD model. In aged 3xTgAD mice, the investigators overexpressed the NF-κB inhibitory protein A20 predominantly in hippocampal GFAP-positive astrocytes. Astrocytic p65 activation decreased, yet Aβ plaques, soluble and insoluble Aβ, and phosphorylated tau increased. APP and its cleavage products were not altered, arguing against increased Aβ production as the explanation. Astrocytic NF-κB inhibition also reduced aquaporin−4 (AQP4), a water-channel protein important for astrocyte polarization and glymphatic solute handling (Jong Huat et al., 2024). These findings suggest that once AD-like pathology is established, at least some NF-κB-dependent astrocytic responses contribute to the handling or containment of pathological proteins.

These two results are not necessarily contradictory. Sustained NF-κB activity can disrupt a previously healthy astrocyte, while disease-associated astrocytic NF-κB may simultaneously induce compensatory functions that become useful once Aβ and tau are already present. Importantly, the study did not follow the same astrocytic manipulation continuously from a healthy state through successive stages of AD. It therefore demonstrates context dependence more convincingly than a defined temporal switch from “protective” to “harmful.”

A distinct mechanism involving extracellular Aβ degradation further demonstrates why the term “amyloid clearance” is too broad. Sudo et al. investigated kallikrein-related peptidase 7 (KLK7), a secreted protease capable of degrading extracellular Aβ. In cultured cells, 24-hour treatment with the NF-κB inhibitors IKK−16 at 2 µM or JSH−23 at 30 µM increased KLK7 expression and enhanced degradation of extracellular Aβ40. Predominantly astrocytic primary mouse glial cultures showed the same direction of effect. Importantly, the reduction in extracellular Aβ could not be explained by increased cellular uptake or loss of cell viability, supporting increased proteolytic degradation as the relevant mechanism. The investigators then tested this mechanism in vivo. IKK−16 was infused directly into one hippocampus at 4 µL of 100 µM solution, delivered at 0.3 µL/min, while the contralateral hippocampus received vehicle. Klk7 expression increased after 24 h in wild-type mice. In 14–16-month-old App^NL-G-F^ knock-in mice, 48 h of local NF-κB inhibition reduced soluble and SDS-insoluble Aβ and decreased plaque size, although plaque number did not change. Most importantly, the Aβ-lowering effect disappeared in App^NL-G-F^ mice lacking Klk7. This knockout experiment strongly supports the sequence NF-κB inhibition → KLK7 upregulation → enhanced extracellular Aβ degradation, rather than KLK7 merely changing in parallel with Aβ (Sudo et al., 2026).

The Sudo et al., study (Sudo et al., 2026) appears at first to conflict with the APP23 experiment, in which astrocytic NF-κB activation reduced compact plaques. In fact, the two studies examine different components of Aβ biology. Astrocytic NF-κB may facilitate glial organization around established plaques while simultaneously suppressing KLK7-dependent proteolysis of extracellular Aβ. These functions can occur in parallel. A smaller plaque after one intervention and greater extracellular Aβ degradation after another therefore do not imply that NF-κB has a single bidirectional “clearance switch.” Rather, they show that plaque organization, cellular uptake, and extracellular proteolysis are mechanistically distinct processes.

The therapeutic implications of the KLK7 mechanism also require caution. IKK−16 was delivered locally to the hippocampus rather than selectively to astrocytes, and KLK7 is not expressed exclusively by astrocytes. The in-vivo observation period was only 24–48 h, which is insufficient to determine chronic effects on cognition, synaptic function, host defence, or other physiological NF-κB programs. The experiment therefore provides strong mechanistic evidence that NF-κB can restrain KLK7-dependent Aβ degradation, but it does not establish that sustained global NF-κB inhibition would be beneficial in AD.

Evidence for detrimental astrocytic NF-κB is stronger when pathway activation is linked to defined secreted mediators and subsequent neuronal injury. Blanco et al. showed that Aβ1–42 activated NF-κB-dependent COX−2 transcription in primary human astrocytes and astrocytoma cells, increasing prostaglandin E2 (PGE2) production (Blanco et al., 2010b). Deletion of κB-responsive promoter elements reduced COX−2 transcription, providing direct evidence of NF-κB-dependent regulation. Conditioned medium from Aβ-activated astrocytes was toxic to neuronal cells, supporting a pathway in which Aβ-induced astrocytic NF-κB activity contributes to a harmful secretory response.

The functional consequence was demonstrated using astrocyte-conditioned medium. Medium collected from Aβ-activated astrocytes transferred toxicity to neuronal cells, indicating that the harmful effect did not require direct astrocyte–neuron contact. The resulting sequence—Aβ → astrocytic NF-κB → COX−2/PGE2-associated secretion → neuronal injury—provides substantially stronger evidence for a detrimental astrocytic program than the observation of reactive astrocyte markers alone. The use of astrocytoma-derived cells and acute Aβ exposure limits direct extrapolation to aged human astrocytes, but the promoter manipulation and conditioned-medium experiment provide a coherent mechanism.

An even more completely resolved astrocyte-to-neuron pathway involves complement component 3 (C3). Lian et al. demonstrated that Aβ activates astrocytic NF-κB and increases C3 production and release. Astrocyte-derived C3 then acts on neuronal C3a receptors, altering intracellular calcium, dendritic structure, AMPA-receptor trafficking, and neuronal network function (Lian et al., 2015b). Importantly, C3aR antagonism reduced these downstream abnormalities and rescued cognitive impairment in APP-transgenic mice. This rescue experiment provides strong evidence for an astrocytic NF-κB → C3 → neuronal C3aR pathway contributing to synaptic and cognitive dysfunction.

Cell-specific genetic studies support the broader principle that astrocytic NF-κB can amplify neuronal proteotoxicity. In Drosophila models, neuronal expression of proteotoxic proteins activated the NF-κB homolog Relish in astrocyte-like glia. Selective reduction of Relish in these glial cells decreased inflammatory gene expression, delayed neurodegeneration, prolonged survival, and also extended lifespan in flies expressing neuronal human Aβ42 (Li et al., 2018). Although fly glia differ from mammalian astrocytes, the cell-restricted genetic design demonstrates a non-cell-autonomous loop in which neuronal proteotoxic stress activates glial NF-κB, which then contributes to further neuronal damage.

Genetic and metabolic background can also determine how readily astrocytes enter an inflammatory NF-κB state. In isogenic human iPSC-derived astrocytes, APOE4 reduced TAGLN3 expression and produced low-grade inflammation with exaggerated NF-κB responses to subsequent stimulation. TAGLN3 supplementation attenuated this phenotype, and interaction between TAGLN3 and IκBα supported a role for TAGLN3 in restraining NF-κB activity (Arnaud et al., 2022b). These findings suggest that APOE4 alters the baseline regulatory state of astrocytes, making them more susceptible to excessive inflammatory signaling.

Altered lipid handling may create a similar predisposition. FABP7 was increased in Aβ-exposed astrocytes, plaque-associated astrocytes in APP/PS1 mice, and human AD tissue. In human iPSC-derived astrocytes, FABP7 overexpression induced a broad inflammatory transcriptional program and activated NF-κB, whereas a ligand-binding-deficient FABP7 mutant failed to reproduce the same response (Hamilton et al., 2024a). This indicates that FABP7-mediated lipid binding is required for the inflammatory phenotype and links astrocytic lipid metabolism to NF-κB-dependent reprogramming. Together, the APOE4 and FABP7 studies suggest that astrocytes exposed to similar pathological stimuli may respond differently according to their genetic and metabolic state.

Among the strongest mechanistic evidence for detrimental astrocytic NF-κB signaling comes from studies of histone deacetylase 7 (HDAC7). Ye et al. found that HDAC7 was selectively increased in astrocytes near amyloid plaques in APP/PS1 mice (Ye et al., 2026). Mechanistically, Aβ-induced HDAC7 interacted with and deacetylated IKKα and IKKβ, promoting IKK activation, NF-κB nuclear translocation, and expression of a neurotoxic astrocytic program. Astrocyte-specific HDAC7 overexpression was sufficient to induce neurotoxic transcription, neuronal loss, and cognitive impairment, including in wild-type and relatively young disease-model mice. Conditioned-medium experiments further showed that the neuronal toxicity was mediated by astrocyte-secreted factors rather than requiring direct cell contact. The reverse manipulations strengthened the causal interpretation. Astrocyte-specific HDAC7 knockdown reduced the neurotoxic phenotype, while IKK inhibition prevented the effects of HDAC7 overexpression, placing IKK–NF-κB downstream of HDAC7. Pharmacological inhibition with TMP195 likewise attenuated IKK–NF-κB signaling, reduced neurotoxic reactive astrocytes, limited neuronal and synaptic loss, and improved behavioral deficits in APP/PS1 mice. The resulting sequence—Aβ → astrocytic HDAC7 → IKKα/IKKβ regulation → NF-κB activation → neurotoxic secretome → synaptic and neuronal loss → cognitive impairment—is among the most complete mechanistic demonstrations of detrimental astrocytic NF-κB signaling in the current AD literature (Ye et al., 2026).

Taken together, these studies show that the apparent dual role of astrocytic NF-κB does not arise because the same biological process inexplicably becomes both beneficial and harmful. Rather, NF-κB controls different astrocytic operations, some of which may be compensatory while others are clearly pathogenic. Astrocytic NF-κB can influence glial organization around established amyloid deposits and may support disease-associated functions such as AQP4 expression. At the same time, it can suppress KLK7-dependent extracellular Aβ degradation and activate COX−2/PGE2, complement C3, and HDAC7-dependent secretory programs that adversely affect neurons. APOE4-dependent TAGLN3 loss and FABP7-dependent lipid signaling further determine how strongly astrocytes enter these inflammatory states.

This distinction also explains why apparently conflicting amyloid findings should not be interpreted from plaque burden alone. A reduction in plaque size could reflect altered glial organization or plaque remodeling, whereas a reduction in soluble and insoluble Aβ after NF-κB inhibition may result from increased extracellular KLK7-mediated proteolysis. Conversely, suppression of astrocytic NF-κB during established disease can remove compensatory processes and accelerate Aβ and tau accumulation. Plaque compaction, extracellular Aβ degradation, soluble Aβ abundance, glial coordination, and neuronal toxicity are distinct outcomes and can move in different directions after the same pathway is manipulated.

The available evidence consequently supports a more selective therapeutic interpretation. Astrocytic NF-κB should not be globally suppressed, because some NF-κB-dependent responses may help the diseased brain manage existing pathology. However, defined neurotoxic outputs have increasingly strong causal support, particularly the COX−2/PGE2, C3/C3aR, and HDAC7–IKK–NF-κB pathways. Therapeutic strategies should therefore aim to disrupt these pathological astrocytic programs while preserving homeostatic and compensatory functions required for protein handling, neurovascular support, and neuronal maintenance.

## 7 Neurovascular NF-κB Signaling: Blood–Brain Barrier (BBB) Dysfunction, Hypoperfusion, and Impaired Aβ Efflux

The neurovascular evidence presents a more consistent picture than the neuronal or glial literature. In AD-related models, NF-κB activation in pericytes and cerebral endothelial cells is predominantly associated with blood–brain barrier (BBB) disruption, increased vascular permeability and leukocyte trafficking, reduced cerebral blood flow, and impaired Aβ efflux. This does not imply that physiological vascular NF-κB signaling is inherently harmful, because its normal roles in host defence, vascular repair, and homeostasis were not directly addressed in these studies. Rather, the available evidence specifically implicates disease-conditioned NF-κB signaling in neurovascular dysfunction.

The strongest evidence involves the interaction between APOE4 and brain pericytes. Bell et al. showed that mice expressing human APOE4, as well as Apoe-deficient mice, developed BBB disruption through activation of a pericytic cyclophilin A (CypA)–NF-κB–matrix metalloproteinase 9 (MMP9) pathway, whereas APOE2 and APOE3 maintained greater pathway restraint (Bell et al., 2012). Astrocyte-derived APOE3 suppressed this signaling through lipoprotein receptors, but APOE4 failed to do so. Increased CypA–NF-κB activity enhanced MMP9-mediated degradation of basement-membrane and tight-junction components, increasing BBB permeability and reducing cerebral blood flow. Importantly, these vascular abnormalities preceded detectable neuronal and synaptic dysfunction, and genetic or pharmacological inhibition of CypA attenuated both vascular and neural abnormalities. The findings therefore support a sequence in which APOE4-dependent loss of restraint on pericytic CypA–NF-κB–MMP9 signaling produces BBB and microcirculatory dysfunction that can precede neuronal injury.

Aβ can also activate NF-κB directly in cerebral endothelial cells. Gonzalez-Velasquez et al. found that soluble Aβ1–40 aggregates produced stronger NF-κB nuclear translocation than monomeric or fibrillar Aβ and caused increased endothelial permeability, monocyte adhesion, and transendothelial migration (Gonzalez-Velasquez et al., 2011). NF-κB inhibition prevented these abnormalities, supporting a direct link between soluble Aβ-induced NF-κB activation and loss of endothelial barrier function. The greater activity of soluble aggregates also emphasizes that vascular effects depend on Aβ assembly state rather than simply total peptide concentration, although the endothelial monoculture used in this study does not reproduce the full complexity of the neurovascular unit.

A complementary endothelial pathway may impair removal of Aβ from the brain. Park et al. showed that Aβ1–42 reduced expression of P-glycoprotein (P-gp), an endothelial transporter involved in brain-to-blood Aβ efflux, in brain endothelial cells and near plaques in 5xFAD mice (Park et al., 2014). Mechanistically, Aβ activated the receptor for advanced glycation end products (RAGE) and NF-κB; RAGE blockade or NF-κB inhibition attenuated P-gp loss, whereas RAGE overexpression enhanced it. These findings support an Aβ → RAGE → NF-κB → P-gp reduction pathway. Such signaling could generate a feed-forward cycle in which increasing Aβ activates endothelial NF-κB, reduced P-gp weakens Aβ efflux, and prolonged cerebral Aβ exposure further sustains vascular dysfunction.

Together, these studies suggest several potentially reinforcing neurovascular mechanisms. APOE4 can weaken the BBB through pericytic CypA–NF-κB–MMP9 signaling, soluble Aβ can increase endothelial permeability and leukocyte trafficking through NF-κB, and Aβ–RAGE–NF-κB signaling can reduce P-gp-mediated Aβ efflux. Barrier disruption may consequently expose neural tissue to blood-derived inflammatory factors, while impaired Aβ efflux prolongs exposure of the neurovascular unit to the initiating pathological stimulus. Although this integrated feed-forward model is biologically plausible, no single study has demonstrated the complete sequence in human AD.

Presenilin 1 (PS1) provides an additional molecular connection between AD-related proteins and endothelial NF-κB signaling. Tanaka et al. showed that PS1 can function independently of its γ-secretase activity as a scaffold for a BCR–CK2α–p65 complex in endothelial cells (Tanaka et al., 2018). This complex promoted phosphorylation of RelA/p65 at Ser529 and increased NF-κB-dependent transcription, whereas Psen1 deficiency reduced p65 phosphorylation and recruitment to target promoters. Unlike the APOE4–CypA–MMP9 and Aβ–RAGE–P-gp pathways, however, the study did not establish consequences for BBB permeability, cerebral perfusion, Aβ transport, or neuronal injury. It therefore identifies an endothelial NF-κB regulatory mechanism whose relevance to AD-associated vascular dysfunction remains unresolved.

Overall, disease-associated neurovascular NF-κB signaling is more consistently linked to detrimental outcomes than the corresponding signaling observed in neurons, microglia, or astrocytes. The strongest evidence connects APOE4-dependent pericytic CypA–NF-κB–MMP9 activity to BBB breakdown and hypoperfusion, while endothelial Aβ–NF-κB and Aβ–RAGE–NF-κB pathways increase permeability, leukocyte trafficking, and potentially impair Aβ efflux (Bell et al., 2012, Park et al., 2014, Gonzalez-Velasquez et al., 2011). These findings do not justify global inhibition of vascular NF-κB, because its physiological functions remain important and incompletely examined in AD models. Instead, they support selective targeting of disease-conditioned vascular pathways, particularly APOE4/CypA–NF-κB–MMP9 and Aβ/RAGE–NF-κB signaling, while preserving normal endothelial and perivascular functions.

## 8 IKKβ in AD: Distinguishing Kinase Functions from NF-κB-Dependent Transcription

The literature on IKKβ in AD appears contradictory because IKKβ activity is often treated as equivalent to canonical NF-κB transcription. Although IKKβ is a major upstream activator of NF-κB, it also regulates autophagy, proteostasis, receptor signaling, and cell-death pathways through mechanisms that are not necessarily mediated by RelA/p65-dependent transcription. Consequently, beneficial or detrimental effects of manipulating IKKβ should not automatically be attributed to NF-κB transcription.

Several acute studies illustrate potentially beneficial functions of IKKβ. In Aβ1–42-exposed mice and HT22 cells, DJ−1 promoted IKKβ activity through von Hippel–Lindau protein (VHL), increased autophagic activity, and reduced phosphorylated tau, whereas suppression of DJ−1 or IKKβ worsened tau-related abnormalities (Chen et al., 2021b). Similarly, bidirectional manipulation of IKKβ in Aβ-treated SH-SY5Y cells and APP/PS1 mice showed that IKKβ activation enhanced autophagy and reduced RIPK1-associated necroptosis, whereas IKKβ silencing produced the opposite effects and increased Aβ accumulation (Wang et al., 2022b). These studies support beneficial IKKβ-dependent effects on proteostasis and cell survival under acute experimental conditions, but neither established that a specific NF-κB dimer or κB-regulated transcriptional program mediated the protection.

The relationship changes in chronic disease models. Schnöder et al. conditionally deleted Ikbkb specifically in neurons of APP- and P301S tau-transgenic mice. Neuronal IKKβ deficiency reduced BACE1 expression and β-secretase activity, cerebral Aβ, phosphorylated tau, apoptosis, and inflammatory abnormalities, while also increasing markers of autophagy (Schnöder et al., 2023). Synaptic preservation and cognitive improvement were evident in the APP model, although cognitive rescue was less apparent in the tau model. These findings show that the relationship between IKKβ and autophagy is context dependent: increasing IKKβ can support autophagy during acute Aβ stress, whereas deleting persistent neuronal IKKβ during chronic amyloid or tau pathology can also improve autophagic markers while reducing disease burden. Thus, IKKβ cannot be assigned a fixed pro- or anti-autophagic role independent of disease state and duration.

Other experimental systems broadly support a detrimental effect of prolonged NF-κB signaling. In Drosophila expressing human Aβ42, genetic reduction of Toll–NF-κB signaling attenuated neurodegeneration, whereas increased signaling worsened the phenotype (Tan et al., 2008b). Similarly, loss of protein arginine methyltransferase 5 (PRMT5) increased E2F1, NF-κB, and GSK3β signaling and promoted neuronal injury in APP-Swedish cells and Aβ-expressing *Caenorhabditis elegans*; inhibition of NF-κB or GSK3β reduced cell death (Quan et al., 2015). These studies support a pathogenic role for prolonged stress-associated NF-κB signaling, although their simplified organisms and cellular models limit direct extrapolation to the human brain.

Pharmacological inhibition provides additional whole-brain evidence. Rangasamy et al. used a NEMO-binding domain peptide to preferentially inhibit inducible IKK/NF-κB activation in 5xFAD mice. Repeated intranasal treatment reduced hippocampal NF-κB activity, microglial activation, inflammatory gene expression, Aβ burden, tau-related abnormalities, neuronal apoptosis, and memory impairment (Rangasamy et al., 2015b). Because the intervention affected multiple brain cell types, it cannot identify the specific cellular or NF-κB complex responsible for the improvement, but it supports a detrimental role for excessive inducible IKK/NF-κB signaling.

The strongest evidence for a defined pathogenic IKKβ–NF-κB program comes from microglial tau studies. In PS19 tauopathy mice, microglia-specific IKKβ activation increased NF-κB-dependent transcription, tau seeding, and anatomical spread, whereas microglial Ikbkb deletion reduced tau propagation, improved chaperone-mediated autophagy, normalized disease-associated microglial states, and improved learning and memory (Wang et al., 2022a). Pharmacological inhibition of IKKβ similarly reduced release of seeding-competent tau from microglia. Notably, NF-κB inhibition increased tau retained within neuronal inclusions while reducing extracellular seeding and anatomical spread, showing that intracellular aggregate burden and propagation are distinct pathological outcomes.

Human postmortem studies provide complementary disease relevance but weaker causal evidence. Activated NF-κB and increased IκB have been detected in regions containing neurofibrillary pathology (Hattori et al., 2001), while phosphorylated p65 has been localized to granulovacuolar degeneration and tau-positive neurites (Yamaguchi et al., 2019b). A nuclear TDP−43–p65 complex was also particularly prominent in a small subset of individuals with MCI and episodic-memory impairment (Ohta et al., 2014). These findings demonstrate that altered NF-κB regulation accompanies tau-associated pathology in human brain tissue, but they cannot determine whether NF-κB activation preceded, followed, or became sequestered within degenerating structures.

Therapeutic studies targeting broader inflammatory networks are consistent with this pattern. Combined inhibition of NF-κB and the NLRP3 inflammasome reduced inflammatory cytokines and glial activation and improved cognitive and tau-related outcomes in aged wild-type and rTg4510 tauopathy mice (Wahl et al., 2024). Because both pathways were targeted simultaneously, however, the specific contribution of NF-κB inhibition cannot be isolated.

Taken together, the apparent contradiction in the IKKβ literature largely disappears when kinase activity is distinguished from NF-κB-dependent transcription. Under acute stress, IKKβ can support autophagy, proteostasis, and survival through mechanisms that may not require a defined canonical NF-κB transcriptional program. In contrast, chronic neuronal IKKβ activity promotes amyloidogenic and tau-related pathology, while microglial IKKβ–NF-κB signaling can directly facilitate the processing and propagation of seeding-competent tau. Autophagy therefore should not be regarded as a fixed downstream consequence of IKKβ activity; its direction depends on cell type, disease duration, upstream stimulus, and the relative contribution of transcriptional and non-transcriptional IKKβ functions. These distinctions are important therapeutically. Broad IKKβ inhibition could suppress pathological RelA-dependent inflammatory or amyloidogenic signaling while simultaneously interfering with beneficial IKKβ-dependent functions in proteostasis and cell survival. A more precise strategy would therefore target the disease-associated receptor, NF-κB dimer, transcriptional cofactor, post-translational modification, or downstream effector responsible for the pathological program. Studies that manipulate IKKβ without demonstrating dimer-specific activation, κB-site dependence, target-gene involvement, or downstream rescue should therefore be interpreted primarily as evidence about IKKβ biology rather than as definitive evidence that canonical NF-κB transcription is protective or harmful.

## 9 Knowledge Gaps and Priorities for Future Research

Despite extensive experimental evidence linking NF-κB to AD, a central question remains unresolved: when, where, and in what molecular form does NF-κB shift from an adaptive response to a disease-promoting program? Protective effects are most consistently observed during transient neuronal stress responses, trophic signaling, c-Rel-dependent antioxidant programs, and selected astrocytic compensatory functions. By contrast, persistent IKKβ–RelA signaling is repeatedly associated with amyloidogenic processing, inflammatory secretion, tau propagation, blood–brain barrier dysfunction, and neuronal injury. It remains unclear whether these patterns represent a temporal transition during disease progression, distinct programs operating simultaneously in different cell types, or both.

Resolving this question will require longitudinal human evidence. Genetic findings such as SHARPIN-associated impaired inducible signaling suggest that abnormalities in NF-κB regulation can precede clinical disease, whereas postmortem studies predominantly reveal altered RelA/p65 activity after substantial pathology has developed. However, these approaches cannot determine whether impaired stress responsiveness in preclinical disease later evolves into persistent inflammatory activation. Longitudinal studies spanning preclinical AD, mild cognitive impairment (MCI), and dementia are therefore needed to relate NF-κB pathway state to amyloid, tau, neurodegeneration, and clinical progression within the same individuals.

Greater cellular and molecular resolution is equally important. Most human studies rely on bulk tissue, conventional immunohistochemistry, or generic markers such as p65 phosphorylation, nuclear translocation, IκB degradation, or κB-DNA binding. These measures cannot distinguish whether signaling originates from neurons, microglia, astrocytes, pericytes, endothelial cells, or infiltrating immune cells, nor can they resolve functionally distinct NF-κB complexes. This distinction is critical because c-Rel-dependent signaling can support neuronal survival, whereas persistent RelA-associated signaling is more often linked to amyloidogenic and inflammatory programs, and p50 can participate in either activating p50/RelA heterodimers or transcriptionally repressive p50/p50 complexes. Future studies should therefore combine spatial transcriptomics, single-nucleus multi-omics, phosphoproteomics, and cell-resolved protein analyses with measurements of dimer composition, promoter occupancy, cofactors, post-translational modifications, and downstream target genes.

The kinetics of signaling also remain poorly defined. Many protective experiments examine NF-κB activation over minutes or hours, whereas AD develops over decades. Acute preconditioning and trophic responses demonstrate that transient activation can be beneficial, but they provide little information about repeated or sustained signaling in the aging brain. Conversely, chronic genetic manipulations frequently reveal detrimental effects but cannot determine whether intermittent physiological NF-κB responses remain necessary. Stage-specific experiments that manipulate the same NF-κB program within the same cell type while independently varying signal intensity and duration are needed to determine whether adaptive signaling crosses a definable threshold into pathology or whether distinct molecular programs are engaged from the outset.

A related methodological issue is the frequent conflation of IKKβ activity with NF-κB-dependent transcription. IKKβ can regulate autophagy, proteostasis, receptor signaling, and cell death independently of canonical NF-κB transcription. This may explain why IKKβ activation can improve autophagic handling or reduce necroptosis in some acute models, whereas persistent neuronal or microglial IKKβ activity promotes amyloid production and tau propagation in chronic disease. Future experiments should combine IKKβ manipulation with dimer-specific blockade, κB-site mutation, target-gene perturbation, and downstream rescue before assigning an observed phenotype specifically to canonical NF-κB signaling.

Important uncertainties also remain within individual cell types. In microglia, uptake of Aβ or tau is often interpreted as successful clearance even though internalization, lysosomal degradation, intracellular retention, extracellular release, and subsequent seeding are biologically distinct processes. Future studies should follow pathological cargo through each of these steps while simultaneously measuring inflammation, neuronal integrity, and functional outcomes. In astrocytes, the key challenge is to distinguish compensatory responses from pathological secretory programs. Astrocytic NF-κB may support plaque-associated organization and selected homeostatic functions while also suppressing KLK7-dependent Aβ degradation or promoting COX−2/PGE2, complement C3, and HDAC7-dependent neurotoxicity. These processes should be measured together rather than inferred from plaque burden or astrogliosis alone.

The neurovascular compartment requires similar integration. Existing studies implicate APOE4-dependent CypA–NF-κB–MMP9 signaling, soluble Aβ-induced endothelial activation, and RAGE–NF-κB-dependent loss of P-glycoprotein in blood–brain barrier disruption, hypoperfusion, leukocyte trafficking, and impaired Aβ efflux. Yet these mechanisms have largely been investigated separately. More physiologically complete human neurovascular models incorporating endothelial cells, pericytes, astrocytic endfeet, basement membrane, physiological shear stress, APOE genotype, and aging-related states are needed to determine how these pathways interact. In parallel, longitudinal measures of BBB integrity and cerebral perfusion should be integrated with amyloid, tau, and inflammatory biomarkers to establish whether neurovascular NF-κB dysfunction precedes or accelerates neuronal degeneration.

Translation is further limited by the heavy reliance on familial AD models, acute Aβ exposure, and relatively young experimental systems. APP/PS1, 5xFAD, APP23, TgCRND8, PS19, and related models have been invaluable for establishing causality but reproduce only selected components of predominantly sporadic late-onset AD. Likewise, high-dose or intracerebroventricular Aβ exposure does not reproduce decades of progressive amyloid accumulation, vascular aging, metabolic dysfunction, and immune adaptation. Human iPSC models reduce species differences but also lose important aging-associated features during reprogramming. Greater use of aged humanized models, directly converted cells that retain aging signatures, multicellular organoid or assembloid systems, and sporadic-risk backgrounds such as APOE4 will therefore be important. Sex should also be incorporated explicitly, because aging, mitochondrial function, immune responsiveness, lipid metabolism, and vascular biology can differ substantially between males and females.

Another major gap is the limited understanding of intercellular NF-κB communication. AD develops within an interconnected neurovascular system rather than within isolated cell populations. Neuronal proteotoxic stress can activate glial NF-κB; astrocytes can modify microglial responses; microglia can promote neuronal injury or tau propagation; astrocytic APOE can regulate pericytic signaling; and endothelial dysfunction can increase exposure of the brain to circulating inflammatory factors. It remains unclear which cellular compartment initiates these interactions, whether the dominant source of pathological NF-κB changes with disease stage, and whether suppressing signaling in one cell type alters pathology in another. Multicellular models allowing independently inducible NF-κB manipulation in neurons, astrocytes, microglia, pericytes, and endothelial cells will be needed to establish causal direction across these networks.

Human genetics may help define such cell-specific NF-κB states. Rare SHARPIN variants suggest that impaired inducible pathway competence can increase LOAD susceptibility, whereas APOE4 promotes exaggerated inflammatory responses in astrocytes and removes restraint from pericytic CypA–NF-κB signaling. These findings imply that genetic risk factors may perturb different components of NF-κB regulation rather than uniformly increasing or decreasing the pathway. Genotype-stratified cellular models and larger genetic studies linked to cell-specific functional assays could determine whether distinct NF-κB endophenotypes identify biologically meaningful subgroups of AD.

A major barrier to addressing these questions in patients is the absence of validated biomarkers of NF-κB state in the living brain. Current human evidence depends largely on genetics, peripheral immune assays, indirect inflammatory markers, or postmortem tissue. None can reliably distinguish preserved inducible signaling from chronic RelA-biased activation or identify the responsible cell type. Development of cerebrospinal-fluid, extracellular-vesicle, molecular-imaging, phosphoproteomic, or transcriptional signatures of specific NF-κB programs would permit longitudinal testing of experimental mechanisms and provide essential pharmacodynamic markers for future intervention studies.

These gaps converge on the principal therapeutic challenge: pathological NF-κB signaling must be suppressed without eliminating physiological functions required for neuronal stress resistance, immune defence, tissue repair, proteostasis, glial support, and vascular homeostasis. The available evidence therefore argues against treating NF-κB as a single drug target. More promising strategies would selectively target disease-conditioned upstream receptors, cell-specific IKK–RelA complexes, pathological cofactors or post-translational modifications, or downstream effectors such as BACE1, C3/C3aR, MMP9, NLRP3, and tau-release pathways. The key objective for future research is therefore not simply to determine whether NF-κB is protective or detrimental, but to identify which NF-κB program is active, in which cell, at which disease stage, and with what biological consequence. Achieving this level of resolution will be essential for moving from global NF-κB modulation toward precision targeting of pathological signaling while preserving its physiological and compensatory functions.

## 10 Conclusion

Current evidence indicates that the apparently opposing roles of NF-κB in AD reflect context-dependent regulation rather than a true biological paradox. Transient and appropriately controlled NF-κB signaling can support neuronal survival, stress adaptation, antioxidant defense, and selected compensatory responses, whereas persistent or disease-conditioned signaling—particularly RelA/p65-dominant activity—is more consistently associated with amyloidogenic processing, chronic neuroinflammation, tau-related pathology, impaired proteostasis, neurovascular dysfunction, and neuronal injury. These effects differ substantially according to cell type, upstream stimulus, signaling duration, dimer composition, and disease state.

Human genetic, postmortem, peripheral, and iPSC-based studies broadly have reinforced this framework, although each provided only a partial view of pathway regulation in the human brain. Importantly, impaired inducible NF-κB responsiveness may increase susceptibility to LOAD, while exaggerated or persistent signaling appears more prominent after pathological processes are established. Whether these states represent sequential phases of disease or distinct parallel forms of dysregulation remains unresolved.

Taken together, the evidence argues against global activation or inhibition of NF-κB as a therapeutic strategy. Future approaches should instead identify and selectively target disease-driving NF-κB programs within specific cellular and molecular contexts while preserving physiological signaling required for homeostasis and adaptive stress responses. Resolving the temporal, cell-specific, and molecular architecture of NF-κB signaling across the AD continuum will be essential for translating this pathway from a broadly implicated inflammatory regulator into a more precise therapeutic target.

## Abbreviations

**NF-κB**: Nuclear factor kappa B; **AD**: Alzheimer disease; **Aβ**: amyloid-β; **IKK**: IκB kinase; **LOAD**: late-onset Alzheimer’s disease; **FABP7**: fatty acid-binding protein 7; **BBB**: blood–brain barrier.

## Ethical Approval and Consent to Participate

Not applicable because this article is a review and did not involve human participants or animals.

## Funding

This work received no specific funding from public, commercial, or not-for-profit funding agencies.

## CRediT authorship contribution statement

**Fatemeh Abutalebian:** Writing – review & editing, Writing – original draft, Investigation. **Hamide Nasiri:** Writing – review & editing, Writing – original draft, Validation, Investigation, Conceptualization. **Ali Azargoonjahromi:** Writing – review & editing, Writing – original draft, Validation, Supervision, Methodology, Investigation, Conceptualization.

## Authors’ Contributions

A.A. conceived and designed the review, supervised the work, drafted the manuscript, undertook critical revision, and prepared and approved the final version. H.N. and F.A. contributed to the investigation and initial drafting of the manuscript, provided critical intellectual revision, and approved the final version.

## Consent for Publication

Not applicable.

## Clinical trial number

Not applicable.

## Declaration of Competing Interest

The authors declare that they have no competing interests.

## Data Availability

Not applicable.
